# Convergent Evolution of Argonaute-2 Slicer Antagonism in Two Distinct Insect RNA Viruses

**DOI:** 10.1371/journal.ppat.1002872

**Published:** 2012-08-16

**Authors:** Joël T. van Mierlo, Alfred W. Bronkhorst, Gijs J. Overheul, Sajna A. Sadanandan, Jens-Ola Ekström, Marco Heestermans, Dan Hultmark, Christophe Antoniewski, Ronald P. van Rij

**Affiliations:** 1 Department of Medical Microbiology, Radboud University Nijmegen Medical Centre, Nijmegen Centre for Molecular Life Sciences, Nijmegen Institute for Infection, Inflammation and Immunity, Nijmegen, The Netherlands; 2 Department of Molecular Biology, Umeå University, Umeå, Sweden; 3 Institute of Biomedical Technology, University of Tampere, Tampere, Finland; 4 Drosophila Genetics and Epigenetics, Université Pierre et Marie Curie Paris VI, CNRS UMR 7622 - Biologie du Développement, Paris, France; Stanford University, United States of America

## Abstract

RNA interference (RNAi) is a major antiviral pathway that shapes evolution of RNA viruses. We show here that Nora virus, a natural *Drosophila* pathogen, is both a target and suppressor of RNAi. We detected viral small RNAs with a signature of *Dicer-2* dependent small interfering RNAs in Nora virus infected *Drosophila*. Furthermore, we demonstrate that the Nora virus VP1 protein contains RNAi suppressive activity *in vitro* and *in vivo* that enhances pathogenicity of recombinant Sindbis virus in an RNAi dependent manner. Nora virus VP1 and the viral suppressor of RNAi of Cricket paralysis virus (1A) antagonized Argonaute-2 (AGO2) Slicer activity of RNA induced silencing complexes pre-loaded with a methylated single-stranded guide strand. The convergent evolution of AGO2 suppression in two unrelated insect RNA viruses highlights the importance of AGO2 in antiviral defense.

## Author Summary

Multi-cellular organisms require a potent immune response to ensure survival under the ongoing assault by microbial pathogens. Co-evolution of virus and host shapes the genome of both pathogen and host. Using *Drosophila melanogaster* as a model, we study virus-host interactions in infections by Nora virus, a non-lethal natural pathogen of fruit flies. Insects depend on the RNA interference (RNAi) pathway for antiviral defense. A hallmark of the antiviral RNAi response is the production of viral small RNAs during infection. We detected Nora virus small RNAs during infection of *Drosophila*, demonstrating that Nora virus is a target of the antiviral RNAi pathway. Furthermore, we show that Nora virus viral protein 1 (VP1) inhibits the catalytic activity of Argonaute-2, a key protein of the RNAi pathway. The 1A protein of Cricket paralysis virus suppresses RNAi via a similar mechanism. Importantly, whereas Nora virus persistently infects *Drosophila*, Cricket paralysis virus induces a lethal infection. Our findings thus indicate that two distantly related viruses independently evolved an RNAi suppressor protein that targets the Argonaute-2 protein. Altogether, our results emphasize the critical role of Argonaute-2 in insect antiviral defense, both in lethal and persistent infections.

## Introduction

An efficient antiviral immune response is essential for the control or elimination of virus infection and for survival of the infected host. The immune system exerts a strong evolutionary pressure that shapes the genetic makeup of viral pathogens. Indeed, viruses evolved counter-defense mechanisms to evade, suppress or inactivate host immunity. Studying these mechanisms provides important insight in the critical steps of antiviral responses and may uncover novel components and regulators of immune pathways.

Plants, fungi, and invertebrate animals rely on the RNA interference (RNAi) pathway for antiviral defense [1], [2]. The initial trigger of an antiviral RNAi response is the recognition and cleavage of viral double-stranded RNA (dsRNA) into viral small interfering RNAs (vsiRNAs), in insects by the ribonuclease Dicer-2 (Dcr-2). These vsiRNAs act as specificity determinants of the Argonaute-2 (AGO2) containing effector nuclease complex RISC (RNA induced silencing complex). RISC maturation involves a number of sequential steps: loading of the vsiRNA into AGO2, cleavage and elimination of the passenger RNA strand, and 2′-O-methylation of the 3′-terminal nucleotide of the retained guide strand. It is thought that vsiRNA-loaded RISC subsequently cleaves viral target RNA (Slicer activity). The hypersensitivity to viral infections of *AGO2* mutant flies and of *AGO2* knockdown mosquitoes provides genetic support for this hypothesis [3]–[7]. Nevertheless, direct evidence supporting this model, for example by the detection of viral Slicer products, is lacking.

The evolution of viral suppressors of RNAi (VSRs) is a testament to the antiviral potential of the RNAi pathway in plants and insects. Given the central role of dsRNA and siRNAs as initiators and specificity determinants of the RNAi pathway, it is not surprising that many VSRs sequester dsRNA. For instance, the Drosophila C virus (DCV) 1A protein binds long dsRNA and shields it from processing by Dcr-2 [6]. Flock House virus (FHV) B2 displays a dual RNA binding activity: it binds long dsRNA as well as siRNAs, thereby preventing their incorporation into RISC [8]–[10]. Similarly, many plant VSRs display dsRNA binding activities, leading to the hypothesis that dsRNA or siRNA binding is a general mechanism for RNAi suppression [11], [12]. Nevertheless, other mechanisms have been reported [1]. The RNAi suppressive activity of the Cricket paralysis virus (CrPV) 1A protein, for example, relies on a direct interaction with AGO2 [13].

VSRs have been identified in dozens of plant viruses from all major virus families [1]. In contrast, VSRs have thus far been identified in only three insect RNA viruses (FHV, CrPV, and DCV). These VSRs were characterized using genetic and biochemical approaches in the model organism *Drosophila melanogaster*. While these viruses indeed efficiently infect *Drosophila* laboratory stocks and cell lines, DCV is the only natural *Drosophila* pathogen among these three viruses [14], [15]. Although FHV and CrPV have a remarkable broad host range in the laboratory, they were originally isolated from non-Drosophilid host species: the New Zealand grass grub (*Costelytra zealandica*) and field crickets (*Teleogryllus oceanicus* and *T. commodus*), respectively [16]–[19].

Since viral counter-defense mechanisms co-evolve with the antiviral immune responses of the host species, it is essential to characterize a VSR within the correct evolutionary context. We therefore set out to identify an RNAi suppressor in Nora virus, a positive sense (+) RNA virus that persistently infects *Drosophila* laboratory stocks as well as *Drosophila* in the wild [20] (D.J. Obbard, personal communication). The genome organization and phylogeny suggest that Nora virus is the type member of a novel virus family within the order of *Picornavirales* [20]. Here we show that Nora virus VP1, the protein product of open reading frame 1 (ORF1), suppresses RNAi in cell culture as well as in flies. In accordance, VP1 is an RNAi-dependent viral pathogenicity factor. In a series of biochemical assays, we show that both Nora virus VP1 as well as CrPV 1A inhibit Slicer activity of a pre-assembled RISC loaded with a methylated guide strand. The lack of amino acid sequence similarity between CrPV 1A and Nora virus VP1 suggests that their Slicer antagonistic activities resulted from convergent evolution, providing direct support for the critical role of AGO2 Slicer activity in antiviral defense.

## Results

### Nora virus is a target of RNAi *in vivo*

Nora virus is an enteric (+) RNA virus that successfully establishes a persistent infection in flies [20]. The mechanism by which this virus establishes persistent infections is unknown. To determine whether Nora virus is a target for Dcr-2, we analyzed the presence of Nora virus small RNAs in the *w^1118^ Drosophila* strain that is widely used as a recipient strain for transgenesis. We isolated and sequenced 19–29 nt small RNAs from body (abdomen and thorax), thorax and head of adult *w^1118^* flies. Sequence reads that perfectly matched the *Drosophila* genome were annotated and discarded. Of the remaining reads, 396.646 (7,8%, body), 237.265 (10,6%, thorax), and 1.099.496 (7,7%, head) matched the published Nora virus sequence (NC_007919.3), indicating that the *w^1118^* strain was infected by Nora virus (Table 1). As RNA viruses rapidly evolve, viral small RNA sequences may have been missed in this initial matching step. We therefore reconstituted the Nora virus genome through an iterative alignment/consensus treatment of the viral small RNA sequences in our libraries [21]. The reconstituted Nora virus genome (rNora virus) differed at only 3.2% of the nucleotides from the published genome sequence. Aligning small RNAs to the rNora virus genome instead of the published Nora virus sequence resulted in an increased number of viral reads in the three libraries (∼121%, Table 1). We therefore used the reconstituted genome as a reference genome in further analyses.

**Table 1:** Annotation of small RNA sequences in libraries from body (abdomen and thorax), thorax, and head of Nora virus infected *w^1118^* adult flies.

	Body	Thorax	Head
Total library	18.296.275	17.280.520	49.633.458
Match to D. melanogaster*	13.184.119	15.033.831	35.435.546
Unmatched*	5.112.156	2.246.689	14.197.912
Nora virus (NC_007919.3)*	396.646	237.265	1.099.496
Nora virus (reconstituted)*	479.572	291.045	1.329.336

* The number of reads matching the *Drosophila* genome, reads that fail to map to the *Drosophila* genome (unmatched), and reads mapping to the Nora virus genome (isolate Umea 2007) and the reconstituted Nora virus genome are indicated for each library.

**Figure 1:**
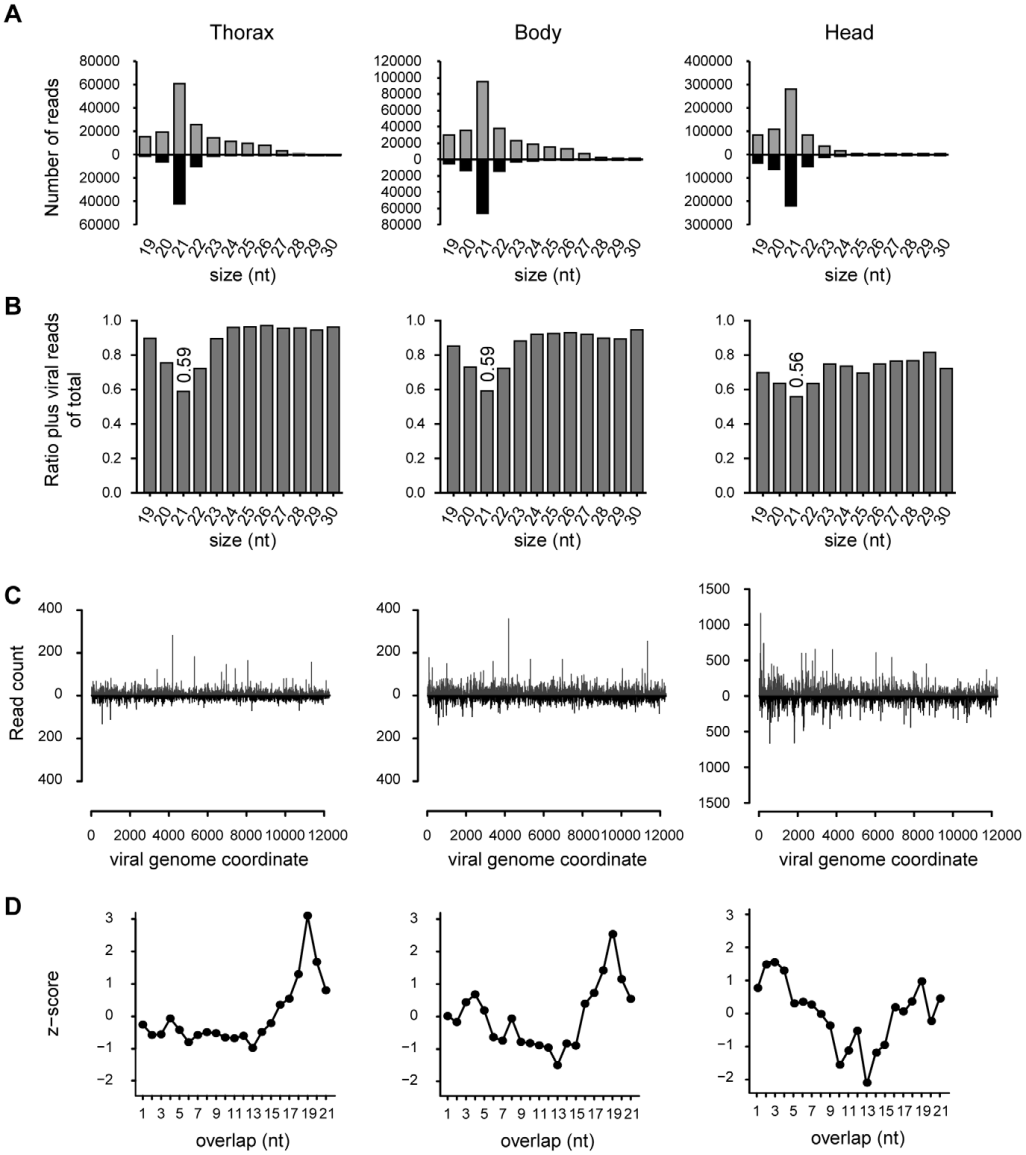
Nora virus is targeted by RNAi in adult flies. (**A**) Size distribution of Nora virus-derived small RNAs in libraries from thoraxes, bodies and heads of *w^1118^* flies. Read counts of small RNAs matching the (+) and (−) viral RNA strands are in gray and black, respectively. (**B**) Proportion of (+) Nora virus small RNA reads of total viral reads. Frequencies were computed from the distributions in panel A for each size class. (**C**) Viral siRNA distribution across the viral genome. The abundance of 21-nt small RNAs matching the (+) and (−) viral RNA strands of the reconstituted Nora virus (rNora) reference genome is shown in gray and black, respectively. (**D**) Z-scores for the number of overlapping pairs of sense and antisense 21-nt Nora virus small RNAs matching the rNora virus reference genome. For each possible overlap of 1 to 21-nt, the number of read pairs was counted and converted into a Z-score.

In all three libraries, Nora virus-derived small RNAs were predominantly 21-nt long, the typical size of Dicer-2 products. The size distribution of small RNAs derived from the (+) RNA strand, however, were noticeably wider than those derived from the (−) RNA strand (Figure 1A). For 21-nt viral RNA reads, there was only a slight bias towards (+) small RNAs (ratio (+) RNA/total RNA ∼0.58), whereas small RNAs of other sizes were predominantly derived from the (+) strand (Figure 1B). In all three libraries, the 21-nt Nora virus-derived RNAs are distributed across the genome, covering both the (+) and (−) viral RNA strands with approximately equal numbers (Figure 1C). These data suggest that dsRNA replication intermediates of Nora virus are processed into 21-nt long siRNAs. The origin of the other size classes of viral small RNAs remains unclear. However, as the predominance of (+) over (−) small RNA reads is reminiscent of the excess of (+) over (−) viral (full-length) RNA that is typically observed in (+) RNA virus infection, they may be due to non-specific RNA degradation.

*Drosophila* Dcr-2 generates 21-nt duplex siRNAs in which 19 nucleotides are base-paired leaving a 2-nt 3′ overhang at each end. For each library, we collected the 21-nt RNA reads whose 5′ ends overlapped with another 21-nt RNA read on the opposite strand of the Nora virus genome. Then, for each possible overlap of 1 to 21-nt, the numbers of read pairs were counted and converted into Z-scores (Figure 1D). This analysis revealed that 21-nt Nora virus-derived RNAs in body and thorax libraries tend to overlap by 19-nt, which is a typical feature of siRNA duplex precursors. This siRNA duplex signature was observed to a lesser extent in head libraries. Very little Nora virus RNA can be detected in the head [22], yet vsiRNA levels were similar in head, thorax, and body (Table 1). The origin of the vsiRNAs in the head and the reason for the less pronounced vsiRNA signature of those small RNAs remain unclear. Altogether, our results strongly suggest that Nora virus double-stranded replication intermediates are processed by Dcr-2 into vsiRNAs that trigger an RNAi response in infected flies.

### Nora virus VP1 suppresses RNAi *in vitro*

**Figure 2:**
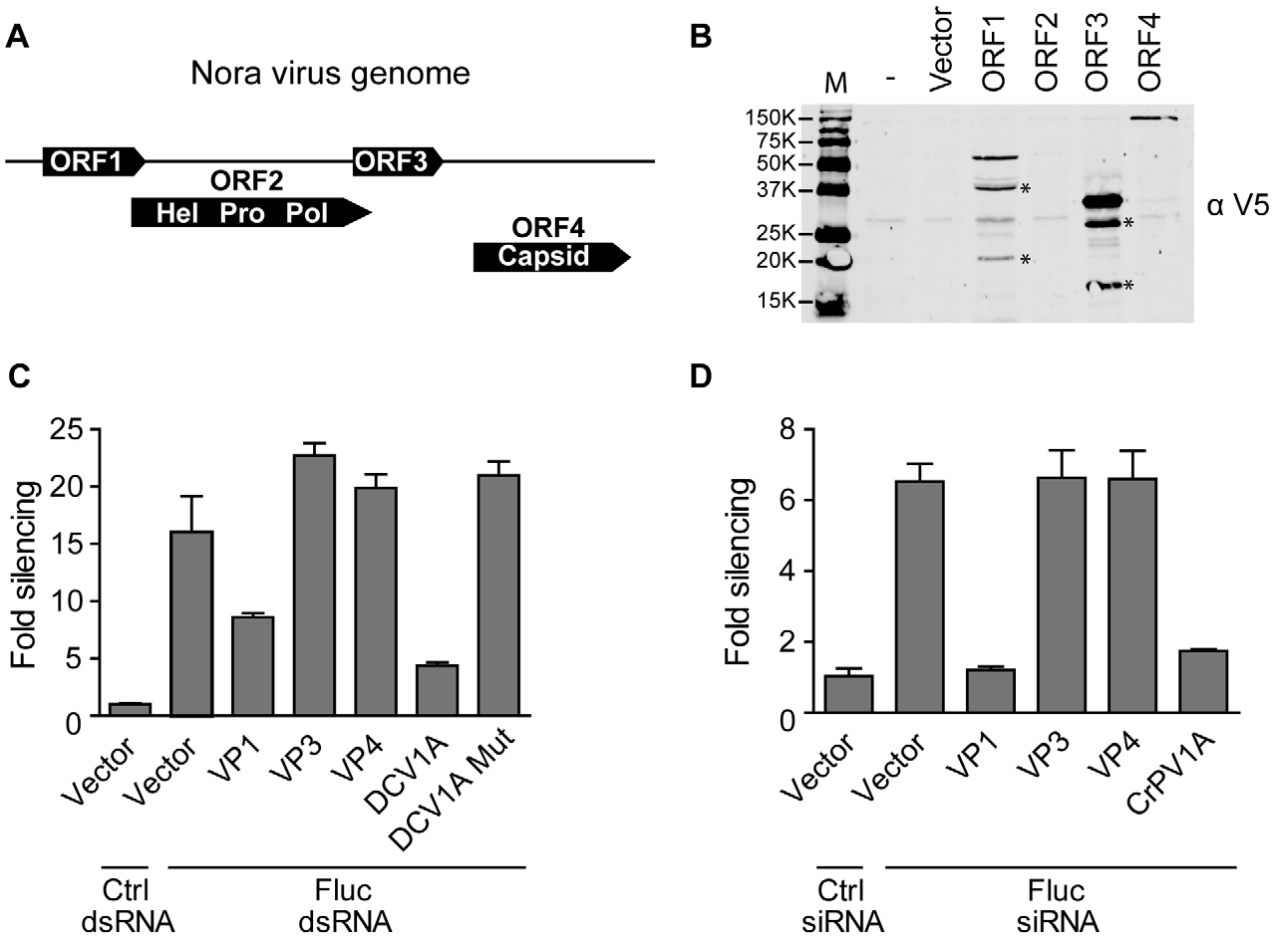
Nora virus VP1 suppresses RNAi *in vitro*. (**A**) Schematic representation of the Nora virus genome with its four predicted ORFs in three different reading frames. There is a 7-nt overlap between ORF1 and ORF2 and a 26-nt overlap between ORF2 with ORF3. An intergenic region of 85 nt separates ORF3 and ORF4. (**B**) Western blot analysis of V5-epitope tagged Nora virus expression constructs. Two days after transfection of the indicated plasmids into S2 cells, expression of the constructs was analyzed by Western blot using the V5 antibody (αV5). Asterisks (*) indicate additional bands that do not correspond to the expected size of the full-length protein product. (**C**) RNAi reporter assay in *Drosophila* S2 cells. Copper-inducible plasmids encoding Fluc and Rluc were transfected into S2 cells together with a construct expressing Nora virus ORF1, 3, and 4, encoding viral protein 1 (VP1), VP3, and VP4, respectively. Two days after transfection, dsRNA targeting Fluc or GFP (Ctrl) was added to the medium. Seven hours later, expression of FLuc and Rluc was induced and luciferase activity was measured the next day. FLuc counts were normalized to Rluc counts and presented as fold silencing relative to the control GFP dsRNA. Plasmids encoding DCV 1A and the K73A mutant (DCV 1A mut) were used as controls. (**D**) siRNA-based RNAi reporter assay. The experiment was performed as described in panel C, but 21-nt Fluc siRNAs were cotransfected with the reporter plasmids to silence gene expression. An siRNA targeting the human MDA5 gene was used as a non-silencing control (Ctrl). Bars in panel C represent averages and standard deviations of five independent samples; bars in panel D represent averages and standard deviations of three independent samples. Panel C and D are representative for two and three independent experiments, respectively.

Our small RNA profiles indicate that Nora virus is targeted by Dcr-2. Nevertheless, the virus efficiently establishes a persistent infection, suggesting that it is able to evade or suppress the antiviral RNAi response. The Nora virus genome contains four open reading frames (ORFs) (Figure 2A). Nora virus ORF2 is predicted to encode the helicase, protease, and polymerase domains that together form a picornavirus-like replication cassette. ORF4 encodes three proteins that make up the Nora virus capsid (VP4A, VP4B, and VP4C) [23]. To determine whether the Nora virus genome encodes an RNAi suppressor, we analyzed the four ORFs in an RNAi sensor assay in *Drosophila* cell culture (Figure 2B–2D). In this assay, S2 cells are transfected with firefly (Fluc) and *Renilla* luciferase (RLuc) reporter plasmids and a plasmid that expresses one of the four viral ORFs. Subsequently, Fluc expression is silenced using specific dsRNA, and Fluc and Rluc activity is monitored. As expected, DCV 1A, a well characterized VSR that binds long dsRNA, efficiently suppressed RNAi, whereas the inactive DCV 1A K73A mutant was unable to do so (Figure 2C and [6]). Cotransfection of the ORF1 expression plasmid also resulted in de-repression of Fluc, suggesting that VP1, the protein product of ORF1, is a suppressor of RNAi. Expression of ORF3 and ORF4 did not affect Fluc activity (Figure 2C). However, since expression of ORF2 and the production of mature capsid proteins from ORF4 were not detectable on western blot, we cannot exclude the possibility that these protein products are able to suppress RNAi as well (Figure 2B).

Next, we tested whether VP1 inhibits the production of siRNAs by Dcr-2 or a subsequent step in the RNAi pathway. To this end, we repeated the RNAi sensor assay using a synthetic siRNA that does not require Dcr-2 cleavage for its silencing activity. Also under these conditions, Nora virus VP1 suppressed silencing of the Fluc reporter. Furthermore, VP1 suppressed RNAi to a similar extent as CrPV 1A, which was previously shown to suppress the effector stage of the RNAi machinery [13] (Figure 2D).

In *Drosophila*, the microRNA (miRNA) and siRNA pathways are separate processes, with Dcr-1 and AGO1 dedicated to the miRNA pathway and Dcr-2 and AGO2 to the siRNA pathway. Nevertheless, crosstalk between the miRNA and RNAi pathways occurs. Using miRNA sensor assays in S2 cells, in which Fluc expression is silenced by endogenous miRNAs or co-expressed primary miRNAs, we observed that VP1 does not suppress miRNA activity (Text S1 and Figure S1). Together, these data indicate that VP1 is able to suppress the RNAi, but not the miRNA pathway, at a step after dsRNA processing by Dcr-2.

### The C-terminus of VP1 is essential for its suppressor activity

**Figure 3:**
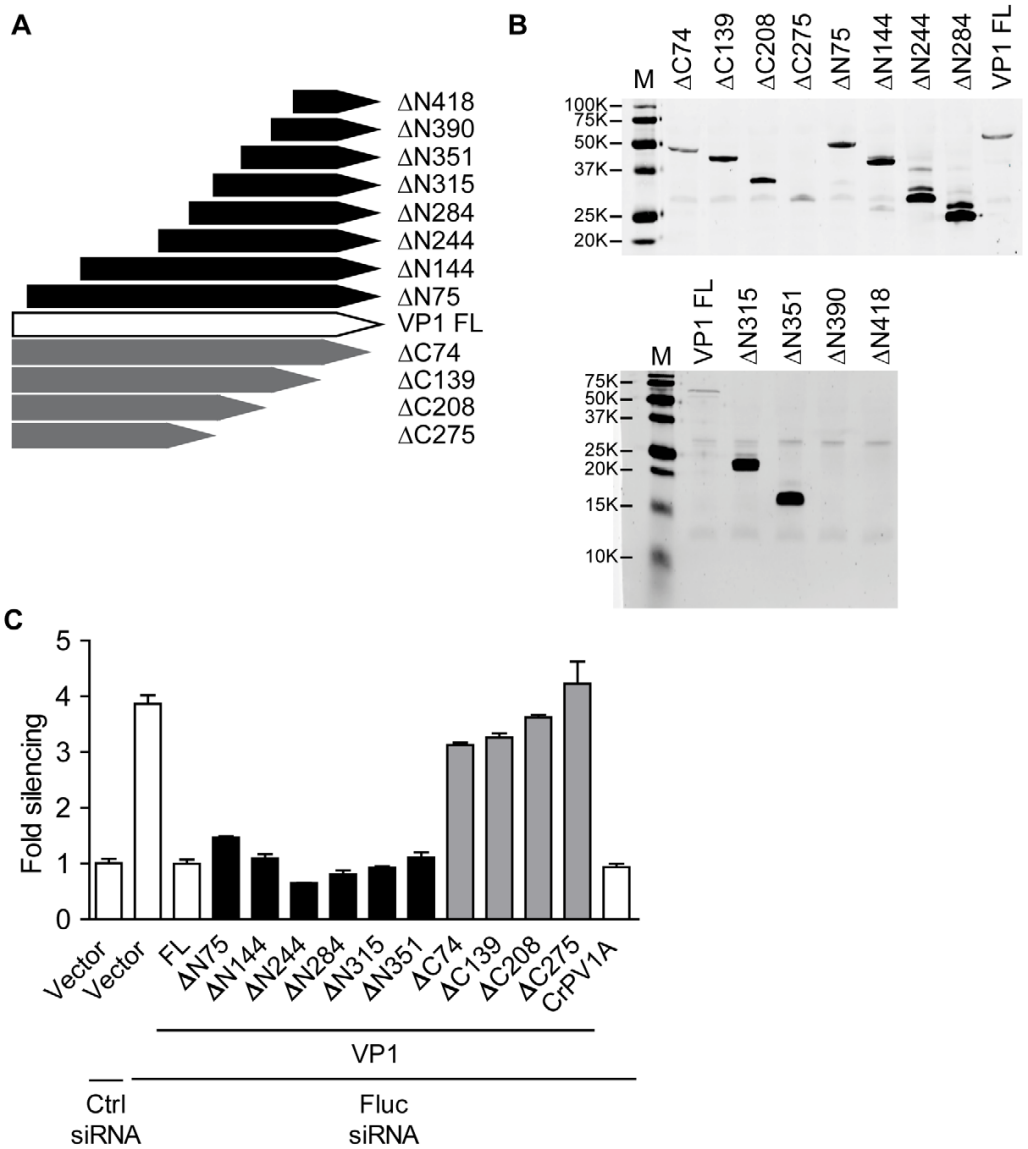
The C-terminus of Nora virus VP1 is essential for RNAi suppressor activity. (**A**) Schematic presentation of full-length (FL) and N- and C-terminal deletion mutants (ΔN and ΔC) of VP1. (**B**) Western blot analysis of VP1 expression constructs. V5 epitope tagged expression constructs were transfected into *Drosophila* S2 cells and expression of VP1^FL^ and the deletion mutants was analyzed by Western blot using a V5 antibody (αV5). (**C**) RNAi reporter assay in S2 cells. The experiment was performed as described in the legend to Figure 2D, using plasmids encoding either CrPV 1A, VP1^FL^ or the VP1 deletion mutants. Bars represent averages and standard deviations of three independent samples. The graph is representative for two independent experiments.

VP1 is highly conserved among different Nora virus isolates (Figure S2). We were unable to predict a protein domain in VP1 suggestive of a mechanism of action. Furthermore, we did not obtain a significant alignment to any other protein from the non-redundant protein sequence database. To map the VSR region of VP1, we generated a series of N- and C-terminal (ΔN and ΔC) truncations and tested them in the RNAi reporter assay in S2 cells (Figures 3A and S3). With the exception of the VP1^ΔN390^ and VP1^ΔN418^ mutants, in which no protein could be detected on Western blot, all VP1^ΔN^ and VP1^ΔC^ constructs produced proteins of the expected size (Figure 3B). Deletion of 74 amino acids (aa) or more from the C-terminus of VP1 resulted in loss of suppressor activity (Figure 3C). This suggests that the active domain of VP1 resides in its C-terminal region. Indeed, deleting up to 351 aa from the N-terminus (VP1^ΔN351^), out of a total of 475 aa, did not affect VSR activity. These results show that the RNAi suppressor activity of VP1 maps to the C-terminal 124 aa.

### VP1 is an RNAi suppressor *in vivo*

**Figure 4:**
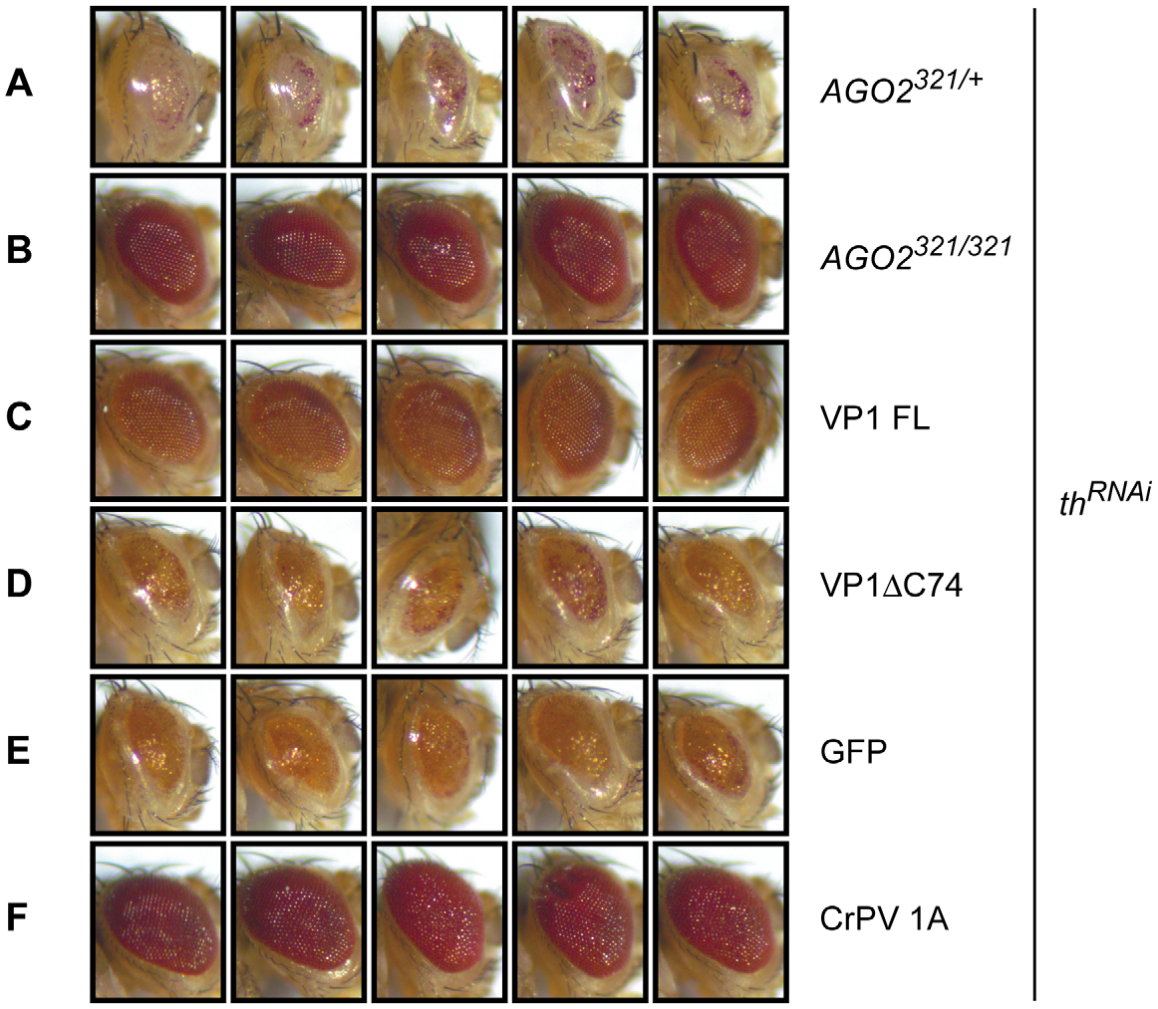
VP1 suppresses RNAi *in vivo*. (**A–F**) RNAi of *Drosophila Inhibitor of Apoptosis1/thread* (*th*) in the eye of adult flies in the indicated genetic background or in the presence of several transgene constructs. RNAi-mediated knockdown of *th* results in a reduced size and pigmentation of the eye and roughening of the eye surface in *AGO2^321^* heterozygotes (**A**), but not in *AGO2^321^* homozygotes (**B**). Eye phenotype of transgenic flies co-expressing the *th* ^RNAi^ construct and Nora virus full-length VP1 (VP1 FL, **C**), a C-terminal deletion mutant of VP1 (VP1^ΔC74^, **D**), GFP (**E**) or CrPV 1A (**F**). Maximum silencing of *th* was examined in the presence of the GFP control transgene (**E**). For each line, five representative pictures of eyes of two- to four-day-old male flies are presented. Pictures are representative for three independent experiments.

We next evaluated the VSR activity of Nora virus VP1 *in vivo* using transgenic flies in which *thread* (*th*), also known as *Drosophila inhibitor of apoptosis 1*, can be silenced by expression of dsRNA targeting this gene (*th^RNAi^* [24], [25]) (Figure 4). Eye-specific expression of *th^RNAi^* using the GMR-Gal4 driver leads to severe apoptosis in the developing eye. As a consequence, *th^RNAi^* flies display a reduced eye size, loss of eye pigmentation, and roughening of the eye surface (Figure 4A, results are shown for *AGO2^321^* heterozygotes; *th^RNAi^* in a wildtype background shows the same phenotype, data not shown and ref. 24). Silencing of *th* in the eye of *th^RNAi^* flies is fully dependent on the RNAi pathway, since the phenotype is lost in an *AGO2* null mutant background (Figure 4B). These results indicate that the *th^RNAi^* sensor fly is a robust system to monitor RNAi activity *in vivo*.

Consistent with its RNAi suppressive activity in cell culture, expression of full-length VP1 (VP1^FL^) in *th^RNAi^* flies resulted in eyes with a normal size and a rescue of the rough eye phenotype (Figure 4C). The phenotype of *th^RNAi^* flies expressing the VP1^ΔC74^ mutant was similar to that of flies expressing GFP as a negative control, confirming that this mutant is functionally inactive (Figure 4D, E). Notably, while VP1 only partially rescued the RNAi-dependent phenotype, CrPV 1A fully reverted the *th^RNAi^* induced phenotype (Figure 4F). Whether this difference is due to a more robust RNAi suppressive activity of CrPV 1A or to a difference in expression level remains to be established.

### VP1 enhances viral pathogenicity *in vivo*

**Figure 5:**
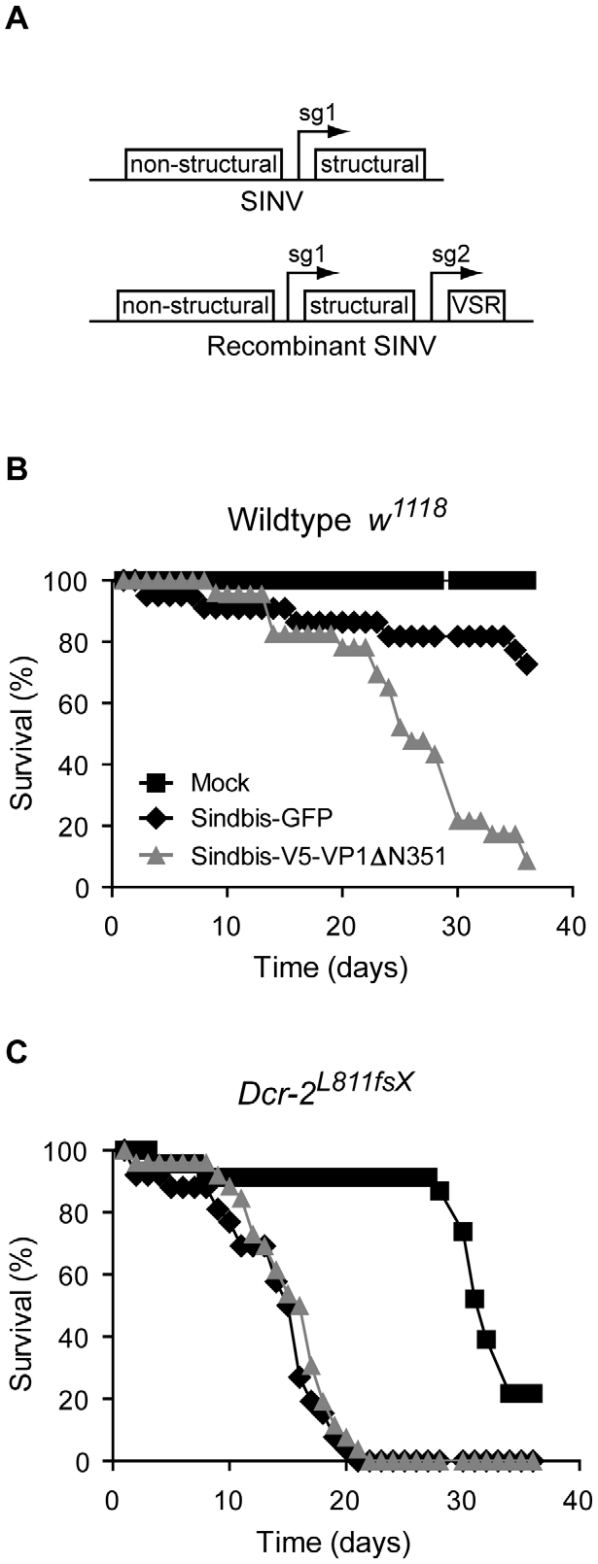
VP1 enhances viral pathogenicity via its RNAi suppressive activity. (**A**) Schematic representation of Sindbis virus (SINV) and SINV recombinant containing a duplicated subgenomic promoter (sg1 and sg2) driving expression of a viral suppressor of RNAi (VSR). (**B** and **C**) Survival curves of *w^1118^* wildtype flies (**B**) and *Dcr-2^L811fsX^* mutants (**C**) infected with SINV recombinants expressing either GFP (black diamond) or VP1^ΔN351^ (gray triangle), or mock infected (black square). Survival curves are representative of two independent experiments.

Having established that VP1 displays RNAi suppressive activity *in vitro* and *in vivo*, we next analyzed the effect of VP1 on viral pathogenicity in adult flies. To this end, we generated recombinant Sindbis virus (SINV) expressing the functional VP1^ΔN351^ (SINV-VP1) or GFP (SINV-GFP) from a second subgenomic promoter (Figure 5A). Although arboviruses are a target of the RNAi pathway during infection in insects [3], [5], [26], we and others have not detected VSR activity in infections with SINV and the related alphavirus Semliki Forest virus [27], [28] (data not shown). Indeed, SINV recombinants expressing the viral RNAi suppressors FHV B2 and CrPV 1A were significantly more pathogenic than their controls in mosquitoes and *Drosophila*, respectively [13], [27].

We injected wildtype *w^1118^* flies with the SINV recombinants and monitored survival over time. SINV-GFP (and the parental SINV virus, data not shown) induced only modest mortality in these flies with a fully functional RNAi response. After 36 days of infection, 73% of the SINV-GFP infected flies and all mock infected flies were still alive. In contrast, SINV-VP1 infection resulted in more severe mortality. SINV-VP1 infected flies died faster and only 9% of the flies survived the 36-days follow up period (Figure 5B). Although these results indicate that VP1 enhances viral pathogenicity, they fail to show that this effect depends on its VSR activity. Viral proteins are often multifunctional and the effect of VP1 on the course of infection might be attributed to another, as yet unknown, activity of VP1. We therefore performed recombinant SINV infections in RNAi deficient *Dcr-2* mutant flies. In this genetic background, an RNAi suppressor is not expected to enhance pathogenicity of the virus. Upon infection with SINV-GFP, the *Dcr-2* mutants died much faster than wild-type flies, confirming that SINV is indeed a target of the RNAi pathway. In contrast to infections in RNAi competent flies, the course of infection of SINV-VP1 and SINV-GFP was remarkably similar in *Dcr-2* mutants, with 100% mortality at 22 days after infection in both cases (Figure 5C). We therefore conclude that VP1 enhances virulence of an RNA virus *in vivo* through its RNAi suppressive activity.

### Nora virus VP1 interferes with the effector phase of RNAi

To further characterize the VSR activity of Nora virus VP1, we next analyzed the activity of VP1 in a series of biochemical assays that monitor individual steps of the RNAi pathway. To this end, we fused the active VP1^ΔN284^ mutant to the maltose binding protein (MBP-VP1) and purified it from *Escherichia coli*. We verified that MBP-VP1 fusion proteins are fully functional in VSR assays in S2 cells to exclude the possibility that MBP interferes with VP1 VSR activity (data not shown).

**Figure 6:**
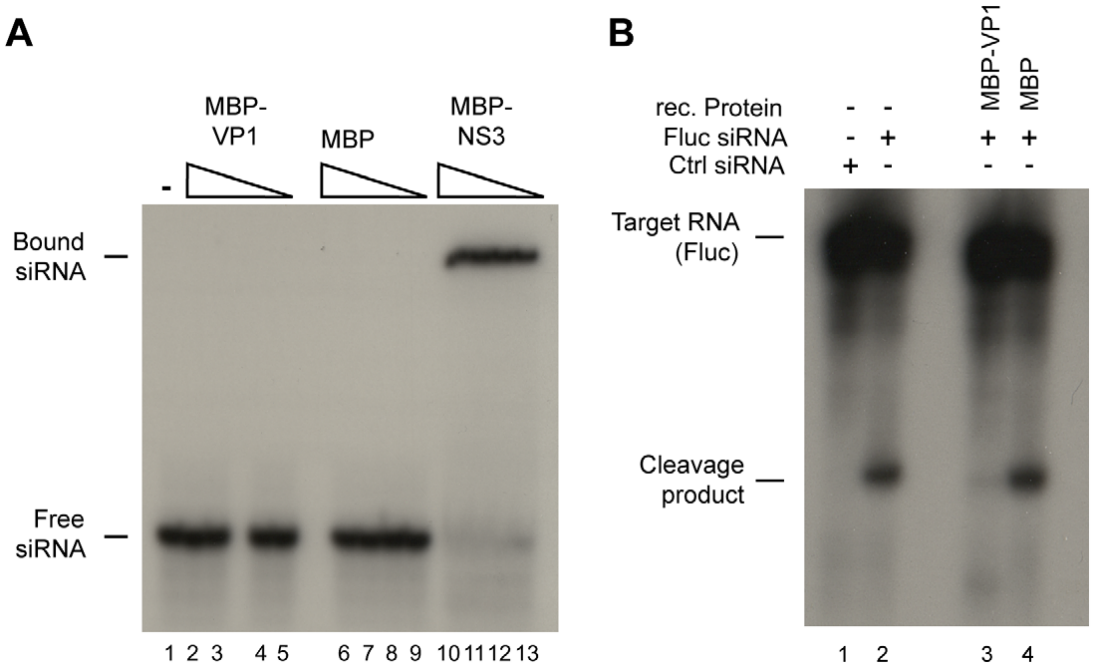
VP1 interferes with the effector phase of the RNAi pathway. (**A**) Mobility shift assays for binding of viral RNAi suppressor proteins to siRNAs. Radiolabeled siRNAs were incubated in buffer (lane 1) or with decreasing amounts of recombinant MBP-VP1^ΔN284^ (lanes 2–5), MBP (lanes 6–9), and MBP-NS3 (lane 10–13). Ten-fold dilutions were used, starting at 2 µM for MBP-VP1^ΔN284^ (lane 2) and 2.6 µM for MBP (lane 6). MBP-NS3 was tested in two-fold dilutions (highest concentration of 8 µM, lane 10). RNA mobility shifts were analyzed on an 8% native polyacrylamide gel. (**B**) RISC Slicer assay in *Drosophila* embryo lysate. Lysates were incubated with non-targeting control siRNA (Ctrl, lane 1) or with Fluc siRNA (lanes 2–4) in the absence (lane 2) or presence of recombinant MBP-VP1^ΔN284^ (lane 3) or MBP (lane 4). RISC cleavage products were analyzed on an 8% denaturing polyacrylamide gel. Slicer assay is representative for two independent experiments.

The ability of VP1 to suppress siRNA-initiated RNAi in S2 cells (Figure 2D) suggests that VP1 inhibits a step downstream of siRNA production by Dcr-2. In accordance, recombinant VP1 was unable to bind long dsRNA in gel mobility shift assays and could not interfere with Dcr-2 mediated processing of long dsRNA into siRNAs in S2 cell extract (Figure S4A, B). We next analyzed whether VP1 is able to bind siRNAs in a gel mobility shift assay. As a positive control, we used a fusion protein of MBP and the Rice hoja blanca virus non-structural protein 3 (NS3), which binds duplex siRNAs with high affinity [29]. Whereas NS3 efficiently bound siRNAs in our assays, we were unable to observe a shift in mobility of siRNAs after incubation with VP1, even at the highest concentrations used (Figure 6A).

Since VP1 is incapable of interfering with the initiator phase of the RNAi pathway, we next examined the effect of VP1 on the effector phase of RNAi. For this purpose, we used an *in vitro* RNA cleavage assay (Slicer assay) in *Drosophila* embryo extract [30], in which a sequence-specific siRNA triggers cleavage of a target RNA. Since the 5′ cap of the target RNA is radioactively labeled, the 5′ cleavage product can be visualized by autoradiography after separation on a denaturing polyacrylamide gel. Indeed, a cleavage product of the expected size was detected if embryo extract was incubated with a target RNA and a specific siRNA. Specific cleavage products were not generated in the presence of a non-specific control siRNA (Figure 6B, lanes 1 and 2). Recombinant VP1 protein, but not control MBP protein, efficiently inhibited the production of cleavage product (Figure 6B, lanes 3 and 4). We note, however, that a minor fraction of the target RNA is still cleaved in the presence of VP1 (Figure 6B, lane 3). Together, these experiments show that VP1 does not affect the initiator phase of the RNAi pathway, but interferes with RISC activity.

### Nora virus VP1 inhibits RISC activity of pre-assembled mature RISC

**Figure 7:**
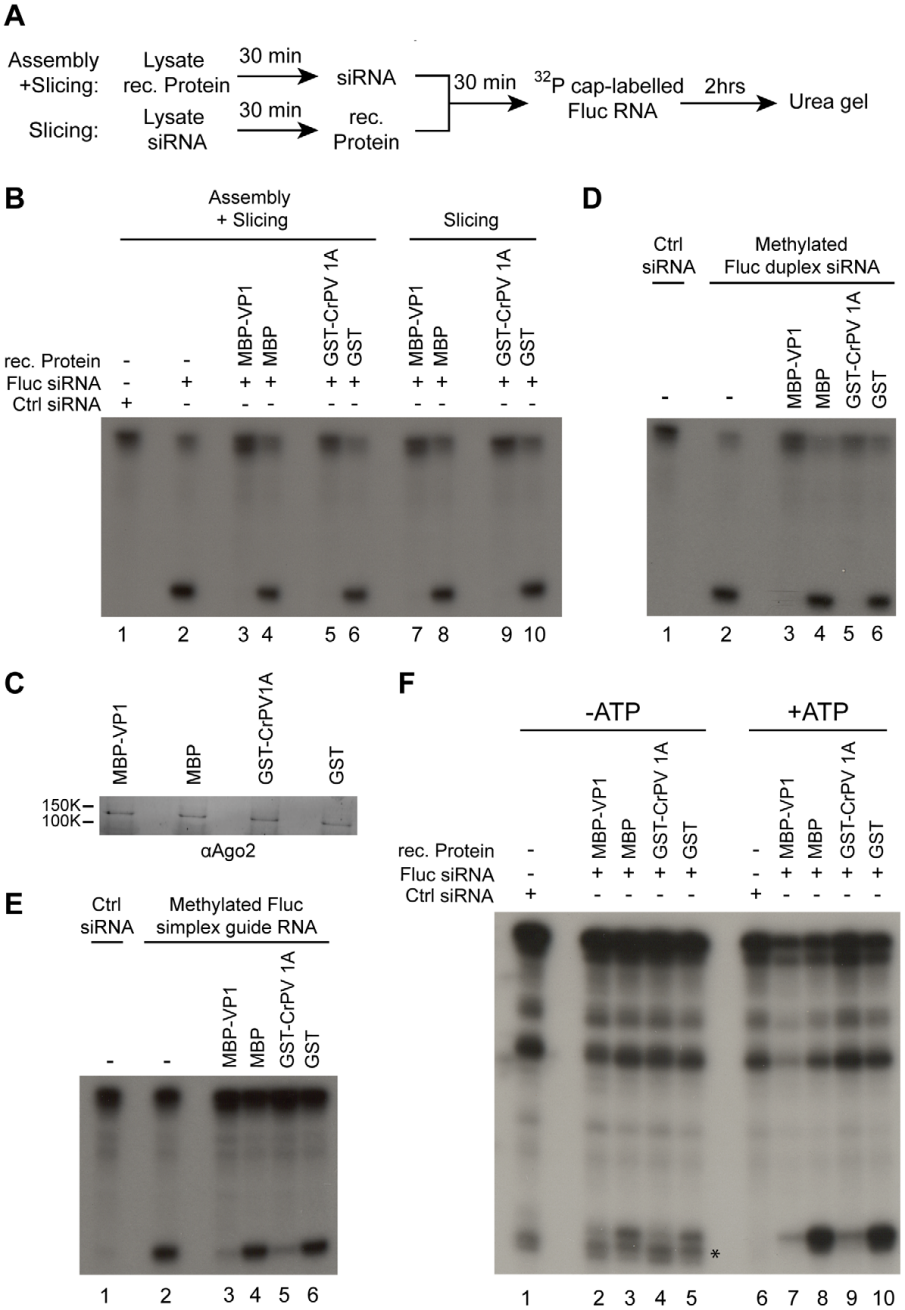
VP1 inhibits Slicer activity of pre-assembled mature RISC. (**A**) Schematic overview of the two experimental conditions of the Slicer assay designed to monitor the effect of recombinant (rec.) proteins on RISC assembly and Slicer activity (top) or on Slicer activity of pre-assembled RISC (bottom) (**B**) Slicer assays in *Drosophila* embryo lysates. RISC activity was analyzed in the presence of a non-targeting control siRNA (lane 1) or a specific Fluc siRNA (lane 2–10). Recombinant proteins were added before (lanes 3–6) or after (lanes 7–10) assembly of RISC as indicated. As a control for possible buffer effects, recombinant protein was substituted by protein storage buffer (lanes 1 and 2). (**C**) Western blot showing the endogenous AGO2 protein levels in embryo lysate after incubation for 2 hours with the indicated recombinant proteins. The blot was developed with AGO2 antibody 4D2. (**D**) Slicer assay using an siRNA with a 2′-O-methylated guide strand. A non-modified control siRNA (lane 1) or a Fluc siRNA duplex containing a 2′-O-methyl group at the 3′ terminal nucleotide of the guide strand (lanes 2–6) was added to embryo lysate 30 minutes prior to the addition of the indicated recombinant proteins. (**E**) Slicer assay using a 2′-O-methylated simplex guide RNA. A control siRNA duplex (lane 1) or a single-stranded Fluc specific guide strand with a 2′-O-methyl group at the 3′ terminal nucleotide (lane 2–6) was added prior to the addition of the indicated recombinant proteins. (**F**) Slicer assays in the presence or absence of ATP. Embryo lysate was incubated with a control siRNA (lanes 1 and 6) or a specific Fluc siRNA (lanes 2–5 and 7–10). ATP was then depleted (lanes 1–5) or depleted and subsequently regenerated (lanes 6–10) and Slicer activity was monitored. An asterisk (*) indicates a non-specific band appearing in RISC assays under ATP depleted conditions.

To discriminate between RISC assembly and target RNA cleavage by a pre-assembled RISC complex, we performed Slicer assays under two experimental conditions (Figure 7A). In the first approach, a purified suppressor protein is added 30 minutes before the siRNA, which allows us to analyze the effect of the VSR on both RISC loading and target cleavage. In the second approach, the embryo extract is incubated with siRNAs for 30 minutes before addition of recombinant protein. This second protocol allows a mature RISC to form prior to the addition of a VSR, thereby allowing us to assess the effect of the VSR on slicing only. As CrPV 1A was previously shown to affect the effector phase of the RNAi pathway [13], we generated recombinant GST-CrPV 1A as well as control GST. These proteins were included in our assays.

Using the first protocol, cleavage of the target RNA was suppressed by VP1 (Figure 7B, lane 3). Strikingly, VP1 was also able to inhibit target cleavage when added to an embryo lysate containing pre-loaded RISC (Figure 7B, lane 7). The observed suppression of slicing was VP1 specific, since MBP alone did not inhibit RNA cleavage (lane 4 and 8). Recombinant CrPV 1A also suppressed slicing in both experimental procedures (Figure 7B, lanes 5 and 9).

To determine if VP1 affects the protein stability of AGO2, we incubated the recombinant proteins in *Drosophila* embryo extract and analyzed endogenous AGO2 protein levels by Western blot. Neither VP1 nor CrPV 1A affected AGO2 protein levels in embryo lysate, indicating that these two proteins do not mediate RNAi suppression through degradation of AGO2 (Figure 7C).

To further confirm the inhibitory effect of VP1 on Slicer activity rather than RISC assembly, we performed Slicer assays using different siRNA guides. During RISC maturation, guide strands in AGO2 are 2′-O-methylated at their 3′ terminal nucleotide by the *Drosophila* methyltransferase Hen1 [31]. This modification protects AGO2 associated siRNAs from degradation by trimming and tailing events that occur when there is extensive base-pairing of the guide RNA with a target RNA [32]. To overcome a requirement for Hen1, an siRNA bearing a 2′-O-methylated 3′-terminal nucleotide on the guide strand was used in Slicer assays. Similar to the non-methylated siRNA, the methylated siRNA produced a specific cleavage product of the expected size (Figure 7D, lane 2). Both Nora virus VP1 and CrPV 1A inhibited the cleavage activity of RISC that was pre-loaded with the methylated siRNA (Figure 7D, lane 3 and 5). Again, the GST and MBP control proteins were unable to affect Slicer activity (Figure 7D, lane 4 and 6).

After loading of the siRNA as a duplex, AGO2 cleaves the passenger strand which is then degraded by the C3PO nuclease complex [33]. To circumvent canonical loading of RISC, we induced RISC formation with a single-stranded methylated guide RNA. Although less efficient, loading of single-stranded guide strands into AGO2 is possible via a bypass mechanism [34], [35]. Indeed, at high concentrations, methylated single-stranded guide RNA induced specific cleavage of cap-labeled target RNA (Figure 7E, lane 2). Interestingly, single-stranded guide RNA-induced target cleavage was specifically inhibited both by Nora virus VP1 and by CrPV 1A (Figure 7E, lanes 3 and 5). These results indicate that both CrPV 1A and Nora virus VP1 inhibit Slicer activity of mature RISC rather than RISC assembly.

Following maturation, RISC binds, cleaves, and releases complementary target RNA, and returns to a Slicer-competent state. *Drosophila* RISC is a multiple turnover complex, in which release of the cleaved target RNA is a rate-limiting step that is greatly enhanced by ATP [36]. We therefore analyzed suppression of Slicer activity under ATP-limiting conditions with a 20-fold molar excess of siRNA over target RNA. RISC was loaded in the presence of ATP, after which creatine kinase was inactivated by NEM, and ATP was depleted (−ATP) by addition of hexokinase and glucose (Figure S5). In parallel, ATP levels were restored (+ATP) after NEM treatment by adding back creatine kinase, and omitting hexokinase treatment. As expected, RISC shows a lower cleavage rate in –ATP conditions than in +ATP conditions (Figure 7F, compare lanes 3 and 5 with lanes 8 and 10). Even under –ATP conditions, Nora virus VP1 and CrPV 1A were able to inhibit Slicer activity (Figure 7F, lanes 2 and 4), suggesting that these two VSRs inhibit the catalytic target cleavage by AGO2.

## Discussion

The mechanisms by which RNA viruses evade sterilizing immunity and establish chronic persistent infections remain poorly understood [37]. Nora virus successfully establishes a persistent infection in *Drosophila*, providing an excellent model to study mechanisms of persistence. We show here that Nora virus is a target of the antiviral RNAi machinery and that it encodes a potent suppressor of RNAi. Of note, Nora virus RNA levels are unaffected by mutations in the RNAi pathway [38]. These observations therefore suggest that dynamic interactions between the antiviral RNAi response and viral counter-defense mechanisms determine viral persistence.

The production of viral siRNAs is a hallmark of an antiviral RNAi response. By detection of Nora virus-derived vsiRNAs in infected fly stocks, we provide direct evidence that Nora virus is a target of Dcr-2. Nora virus vsiRNAs are distributed across the viral genome, with similar amounts derived from the (+) and (−) RNA strands. During (+) RNA virus infection, (+) viral RNA accumulates in large excess over (−) viral RNA (∼50–100 fold). Cleavage of structured RNA elements by Dcr-2 is therefore expected to produce viral small RNAs that mirror this asymmetric distribution. Thus, similar to other RNA viruses, our results imply that Dcr-2 targets the dsRNA intermediates in Nora virus replication [2], [4], [39]–[41].

The current model proposes that the antiviral RNAi response relies on dicing of viral dsRNA and on slicing of viral target RNAs using vsiRNAs as a guide. Genetic analyses support the role of AGO2 in antiviral defense: *AGO2* mutants are hypersensitive to a number of RNA virus infections [3]–[7], [42]. Yet, interpretation of this *AGO2* phenotype is complicated by other cellular functions of AGO2, such as regulation of cellular gene transcription and control of transposon activity [43]–[45]. An alternative model proposes that dicing of double-stranded replication intermediates plays an important role in latent virus infection [46]. Dicing of an essential replication intermediate by Dicer-2 should theoretically be sufficient to abort a productive virus replication cycle. The convergent evolution of VSRs that suppress the catalytic activity of AGO2 in two distantly related RNA viruses, Nora virus and CrPV, underlines the essential role of AGO2 Slicer activity in antiviral defense, also in persistent infections *in vivo*. Importantly, these two viruses display a strikingly different course of infection – CrPV causes a lethal infection, whereas Nora virus establishes a non-lethal, persistent infection – suggesting that the interaction between a VSR and the host RNAi machinery is not the main determinant for viral pathogenicity.

## Materials and Methods

### Small RNA sequencing and analysis

Total RNA was extracted from dissected heads, bodies (abdomens and thoraxes) and thoraxes from *w^1118^* male flies using Trizol reagent (Invitrogen), and RNA quality was verified on a Bioanalyzer (Agilent). Small RNAs were then cloned using the DGE-Small RNA Sample Prep Kit and the Small RNA v1.5 Sample Preparation Kit (Illumina) following the manufacturer's instructions. Libraries were sequenced on the Illumina HiSeq platform.

Sequence reads were clipped from 3′ adapters using fastx_clipper (http://hannonlab.cshl.edu/fastx_toolkit/). Reads in which the adapter sequence (CTGTAGGCACCATCAATCGT) could not be detected were discarded. Only the clipped 19–30 nt reads were retained. Sequence reads were first matched against the *Drosophila* genome (v5.37) using Bowtie (http://bowtie-bio.sourceforge.net/index.shtml). Reads not matching the *Drosophila* genome were then matched against the published Nora virus sequence (NC_007919.3, isolate Umeå 2007), allowing one mismatch during alignment. Viral small RNAs were then used to reconstitute a small RNA-based consensus genome sequence (rNora virus, JX220408) using Paparazzi [21] with NC_007919.3 as a starting viral reference genome. Distributions of Nora virus small RNA sizes were computed by parsing the Bowtie outputs with a python script (available upon request). Small RNA profiles were generated by collecting the 21-nt reads that matched the rNora virus sequence allowing one mismatch, and their frequency relative to their 5′ position in the rNora virus (+) or (−) genomic strand was plotted in R. siRNA duplex signatures were calculated according to an algorithm developed to calculate overlap in piRNA sequence reads [47], [48]. The distribution of siRNA overlaps was computed by collecting the 21-nt rNora virus RNA reads whose 5′ ends overlapped with another 21-nt read on the opposite strand. For each possible overlap of 1 to 21 nt (i), the number of read pairs (O) was counted and converted to a Z-score with the formula Z(i) = (O(i)-mean(O))/standard deviation (O). Small RNA sequences were deposited to the Sequence Read Archive (SRA) at the National Center for Biotechnology Information (NCBI) under accession number SRA054241.

### Cell culture and viruses

*Drosophila* S2 cells were cultured at 25°C in Schneider's medium (Invitrogen) supplemented with 10% heat inactivated fetal calf serum, 50 U/mL penicillin, and 50 µg/mL streptomycin (Invitrogen). DCV was cultured and titered on S2 cells as described previously [6]. For the production of recombinant SINV, the coding sequence of either GFP or the N-terminal V5 epitope tagged VP1^ΔN351^ was cloned into the *Xba*I site of the double subgenomic pTE3'2J vector [49]. The resulting plasmids were linearized by *Xho*I restriction, purified and used as template for *in vitro* transcription using the mMESSAGE mMACHINE SP6 High Yield Capped RNA Transcription kit (Ambion). *In vitro* transcribed RNA was purified using the RNeasy kit (Qiagen) and transfected into BHK cells. Viral titers in the supernatant were determined by plaque assay on BHK cells.

### RNAi reporter assay in S2 cells

RNAi reporter assays were performed as described previously using 25 ng pMT-GL3, 6 ng pMT-Ren, and 25 ng suppressor plasmid per well of a 96-well plate [50]. Plasmids encoding Nora virus cDNA constructs were generated as described in Protocol S1.

### Flies and fly injections

Flies were maintained on standard medium at 25°C with a light/dark cycle of 12 hours/12 hours. Fly stocks that were used for Sindbis virus infection and for preparation of embryo lysate were cleared of *Wolbachia* and endogenous virus infection (see Protocol S1). We used the following fly stocks and alleles: *UAS-CrPV 1A* [13], [51], *AGO2^321^* [52], *Dcr-2^L811fsX^* [53], *th^RNAi^* [24], [25]. The coding sequences of the full-length VP1 and the inactive VP1^ΔC74^ mutant with an N-terminal V5 epitope tag were cloned into the pUAST vector using the *Sac*II and *Xba*I restriction sites [54]. The resulting plasmids were microinjected into *Drosophila w^1118^* embryos to generate transgenic fly lines (Bestgene Inc). Virus infections of adult female flies were performed as described previously using 5,000 PFU of recombinant SINV [6]. Survival was monitored daily. *In vivo* RNAi experiments were performed by crossing *GMR-Gal4*, *UAS-th^RNAi^/CyO* virgins (Meyer et al., 2006) with *UAS-VSR/TM3 Sb* flies. The eye phenotype was monitored in two- to four-day-old male F1 offspring lacking the *CyO* and *TM3 Sb* balancers.

### Production of recombinant proteins in *E. coli*

The GST and MBP fusion proteins were purified from *E. coli* as described in Protocol S1. Purified recombinant proteins were dialyzed against dialysis buffer (20 mM Tris-HCl, 0.5 mM EDTA, 5 mM MgCl_2_, 1 mM DTT, 140 mM NaCl, 2.7 mM KCl) Recombinant proteins were stored as aliquots at −80°C in dialysis buffer containing 30% glycerol.

### Gel mobility shift, Dicer and Slicer assays

Gel mobility shift assays were performed as described [6]. Briefly, uniformly radio-labeled 113 nt long dsRNA (50 cps/reaction) or end-labeled siRNAs (200 cps/reaction) were incubated with purified recombinant protein for 30 minutes at room temperature. Samples were then separated on an 8% native polyacrylamide gel and exposed to a Kodak Biomax XAR film.

Dicer and Slicer assays were performed according to the protocol of Haley and colleagues with minor modifications, described in Protocol S1 [30]. For Slicer assays with the methylated duplex, Fluc guide strand 5′- UCG AAG UAC UCA GCG UAA GU[mU] and passenger strand 5′- CUU ACG CUG AGU ACU UCG AUU were annealed by incubating 20 µM of each siRNA strand in annealing buffer (100 mM potassium acetate, 30 mM HEPES-KOH at pH 7.4, 2 mM magnesium acetate) for 1 min at 90°C, followed by incubation for 1 hour at 37°C. For guide strand loading of RISC, embryo lysates were incubated with Fluc single-stranded guide strand RNA at a final concentration of 10 µM. Radiolabeled probes and target RNA for gel shift and Slicer assays are described in Protocol S1.

## Supporting Information

Figure S1 **VP1 is unable to suppress the miRNA pathway.** A firefly luciferase (Fluc) construct containing the par6 3′UTR, a target for miRNA1 (Fluc-par6), was co-transfected with plasmids encoding *Renilla* luciferase (Rluc) and either Nora virus VP1 or the inactive VP1^ΔC74^ mutant. Fluc-par6 expression was silenced by co-transfecting a plasmid encoding pri-miRNA1, whereas a pri-miRNA12 expressing construct was used as a negative control. *AGO1* or *AGO2* gene expression was knocked down by co-transfection of dsRNA targeting these genes (dsAGO1 and dsAGO2, respectively). Expression of Fluc and Rluc was induced two days after transfection, and reporter activities were measured three days after transfection. Rluc activity was used to normalize Fluc activity within each sample, and data were normalized to the pri-miR12 treated sample. Bars represent averages and standard deviations of biological triplicates. A representative graph of two independent experiments is shown. The numbers represent p-values relative to pri-miR1 treated vector control samples in a two-tailed Student's t-test assuming equal variances.
(TIF)

Figure S2 **Alignment of VP1 sequences from different Nora virus isolates.** Alignment of VP1 sequences of Nora virus isolate Umeå 2007 (accession number GQ257737) and Nora virus sequences from infected fly stocks from our own laboratory (isolates NL1 and NL2, GenBank accession number JQ288019 and JQ288020). We analyzed VP1 sequences in a total of eight Nora virus infected fly stocks. Five VP1 sequences were identical to NL1, one was the NL2 sequence, and two stocks contained a mixed population of Nora virus sequences. These eight stocks were obtained from five different laboratories or stock centers. However, they have been maintained in our laboratory before we tested them for Nora virus infection, and we cannot exclude the possibility that they became infected in our laboratory. Although we therefore cannot infer overall virus diversity from these data, they do indicate that VP1 is a conserved protein. The FR1 isolate is the Nora virus genome that was reconstituted from small RNA sequences from wildtype *w^1118^* flies from a laboratory based in France (GenBank accession number JX220408).
(TIF)

Figure S3 **Nucleotide and protein sequence of full-length and VP1 mutants fused to V5-His tag at the N-terminus.** Nucleotide and amino acid sequence of V5 epitope and Histidine (His) tagged full-length VP1 sequence (VP1^FL^). A linker sequence between the His tag and VP1 was created to facilitate cloning. Start and stop sites of the respective N- and C-terminal deletion mutants of VP1 are indicated. The VP1 deletion mutants were fused to the V5-His tag in an identical way as the VP1^FL^ construct.
(TIF)

Figure S4 **Nora virus VP1 is unable to bind long dsRNA or to interfere with Dcr-2 activity.** (**A**) Mobility shift assay of suppressor proteins with long dsRNA. Uniformly radiolabeled long dsRNA was incubated for 30 minutes with buffer (lane 1) or recombinant MBP-VP1^ΔN284^ (lanes 2–4), MBP (lanes 5–7), GST-DCV 1A (lanes 8–10) or GST (lanes 11–13). Ten-fold dilutions of recombinant protein were used starting from the following concentrations: MBP-VP1^ΔN284^ (2 µM, lane 2), MBP (2.6 µM, lane 5), GST-DCV 1A (1 µM, lane 8), and GST (2.24 µM, lane 11). RNA mobility shifts were analyzed on an 8% native polyacrylamide gel. (**B**) Dicer activity in S2 cell extract in the presence of viral suppressor proteins. Uniformly radiolabeled long dsRNA was incubated in S2 cell extract for 3 hours with buffer (lane 3) or the indicated recombinant proteins. Two-fold dilutions were used for MBP-VP1^ΔN284^ (lanes 4–7, highest concentration 1.1 µM) and MBP (lanes 8–11, highest concentration 4.2 µM). Two independent preparations of GST-DCV 1A were used (lane 12, concentration of 0.54 µM and lane 13, concentration of 0.03 µM). GST was used at a concentration of 1.2 µM (lane 14). As size markers, dsRNA input (lane 1) and end-labelled siRNAs (lane 2) were used. Dicer products were analyzed on a 12% denaturing polyacrylamide gel.
(TIF)

Figure S5 **ATP depletion during Slicer assay.** (**A**) Schematic representation of the protocol used to deplete (−ATP) or to regenerate ATP after initial depletion (+ATP) for Slicer assays of Figure 7F. For RISC loading, *Drosophila* embryo lysate was incubated with an siRNA for 30 minutes under standard conditions. Subsequently, N-ethylmaleimide (NEM) was added in both conditions to inhibit the ATP regenerating activity of creatine kinase. After incubating the reactions for 10 minutes on ice, DTT was added to quench the NEM in both conditions. Hexokinase, glucose, and milliQ water (MQ) were added in the –ATP protocol to deplete the pool of ATP. For the +ATP condition, Hexokinase was substituted by hexokinase buffer, and MQ was substituted for Creatine kinase to restore the ATP regenerating activity. Subsequently, the reactions were incubated for 30 minutes after which recombinant protein (rec. protein) was added. Following another 30 minutes incubation period, the ^32^P-cap-labelled RNA was added to the reaction, after which the incubation was continued for another 2 hours. Subsequently, reactions were analyzed on a polyacrylamide gel. (**B**) ATP concentrations before and after the Slicer assay under –ATP and +ATP conditions. ATP levels were measured at the moment of target RNA addition (0 hrs) or after 2 hours of incubation with target RNA. For ATP concentration measurements, recombinant protein was substituted for protein storage buffer, and target RNA was substituted for MQ. ATP levels were measured using the Celltiter-Glo Luminescent Cell Viability Assay (Promega) according to the manufacturer's protocol.
(TIF)

Protocol S1 **Extended and supplemental methods for molecular cloning, miRNA sensor assay, clearance of** ***Wolbachia*** **and endogenous viruses from fly stocks, production of recombinant proteins in** ***E. coli*** **, production of radio-labeled probes and target RNA, Dicer and Slicer assays.**
(DOC)

Text S1 **Nora virus VP1 is unable to suppress the miRNA pathway.**
(DOC)
