# Supplementary figures and images for: Convergent Evolution of Argonaute-2 Slicer Antagonism in Two Distinct Insect RNA Viruses
Source: PLoS Pathog. 2012 Aug 16;8(8):e1002872. doi: 10.1371/journal.ppat.1002872 (PMC3420963; doi:10.1371/journal.ppat.1002872)

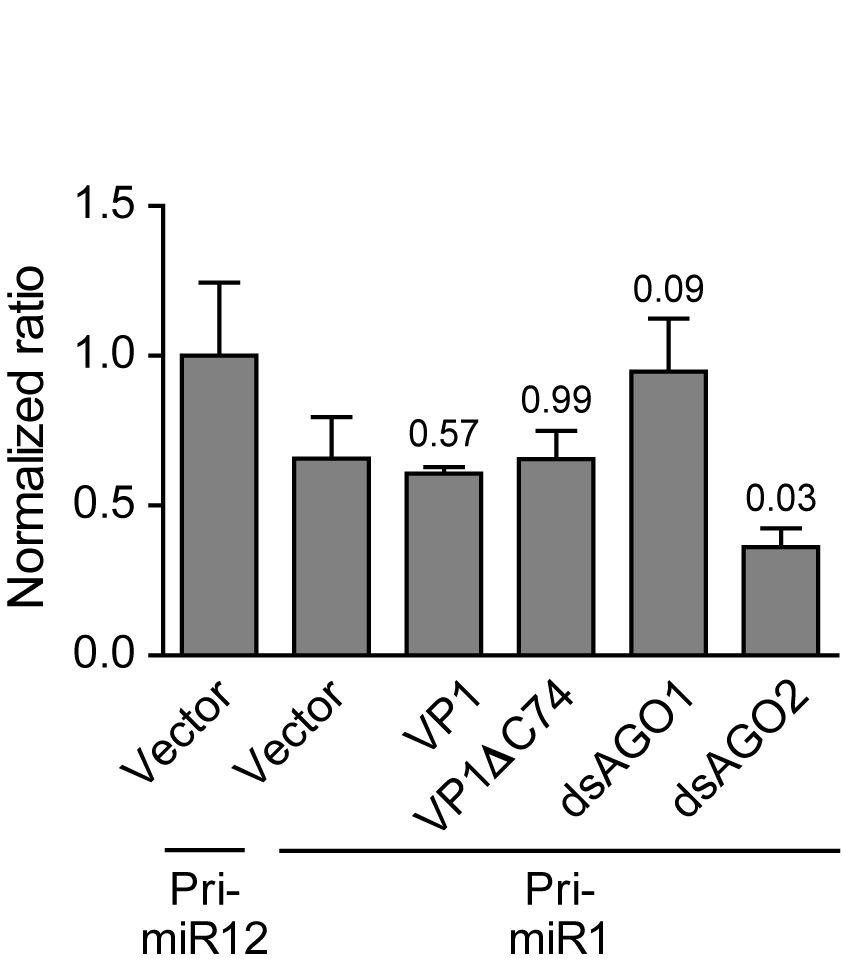

Supplement: Figure S1 — VP1 is unable to suppress the miRNA pathway. A firefly luciferase (Fluc) construct containing the par6 3′UTR, a target for miRNA1 (Fluc-par6), was co-transfected with plasmids encoding Renilla luciferase (Rluc) and either Nora virus VP1 or the inactive VP1ΔC74 mutant. Fluc-par6 expression was silenced by co-transfecting a plasmid encoding pri-miRNA1, whereas a pri-miRNA12 expressing construct was used as a negative control. AGO1 or AGO2 gene expression was knocked down by co-transfection of dsRNA targeting these genes (dsAGO1 and dsAGO2, respectively). Expression of Fluc and Rluc was induced two days after transfection, and reporter activities were measured three days after transfection. Rluc activity was used to normalize Fluc activity within each sample, and data were normalized to the pri-miR12 treated sample. Bars represent averages and standard deviations of biological triplicates. A representative graph of two independent experiments is shown. The numbers represent p-values relative to pri-miR1 treated vector control samples in a two-tailed Student's t-test assuming equal variances. (TIF) [file ppat.1002872.s001.tif]

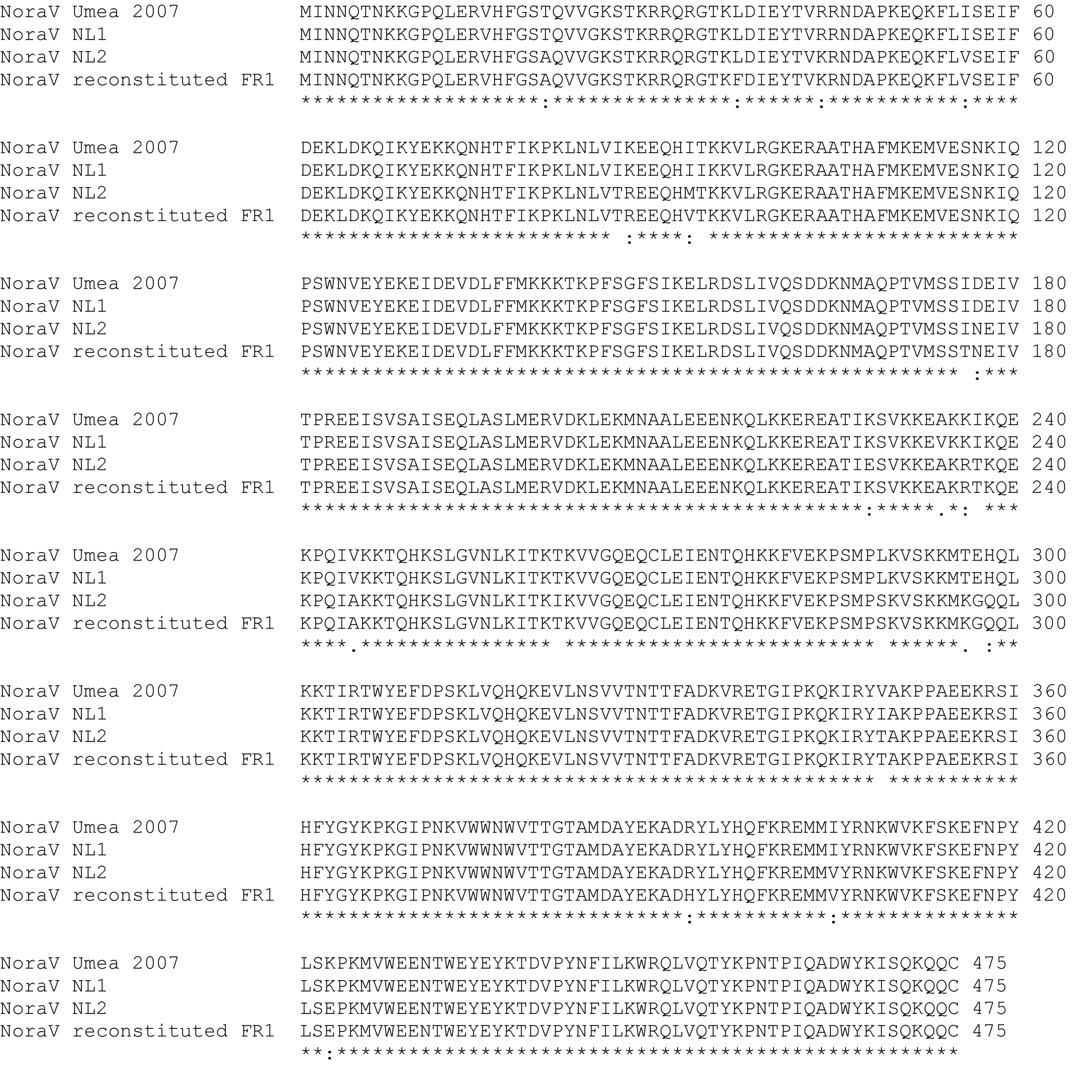

Supplement: Figure S2 — Alignment of VP1 sequences from different Nora virus isolates. Alignment of VP1 sequences of Nora virus isolate Umeå 2007 (accession number GQ257737) and Nora virus sequences from infected fly stocks from our own laboratory (isolates NL1 and NL2, GenBank accession number JQ288019 and JQ288020). We analyzed VP1 sequences in a total of eight Nora virus infected fly stocks. Five VP1 sequences were identical to NL1, one was the NL2 sequence, and two stocks contained a mixed population of Nora virus sequences. These eight stocks were obtained from five different laboratories or stock centers. However, they have been maintained in our laboratory before we tested them for Nora virus infection, and we cannot exclude the possibility that they became infected in our laboratory. Although we therefore cannot infer overall virus diversity from these data, they do indicate that VP1 is a conserved protein. The FR1 isolate is the Nora virus genome that was reconstituted from small RNA sequences from wildtype w1118 flies from a laboratory based in France (GenBank accession number JX220408). (TIF) [file ppat.1002872.s002.tif]

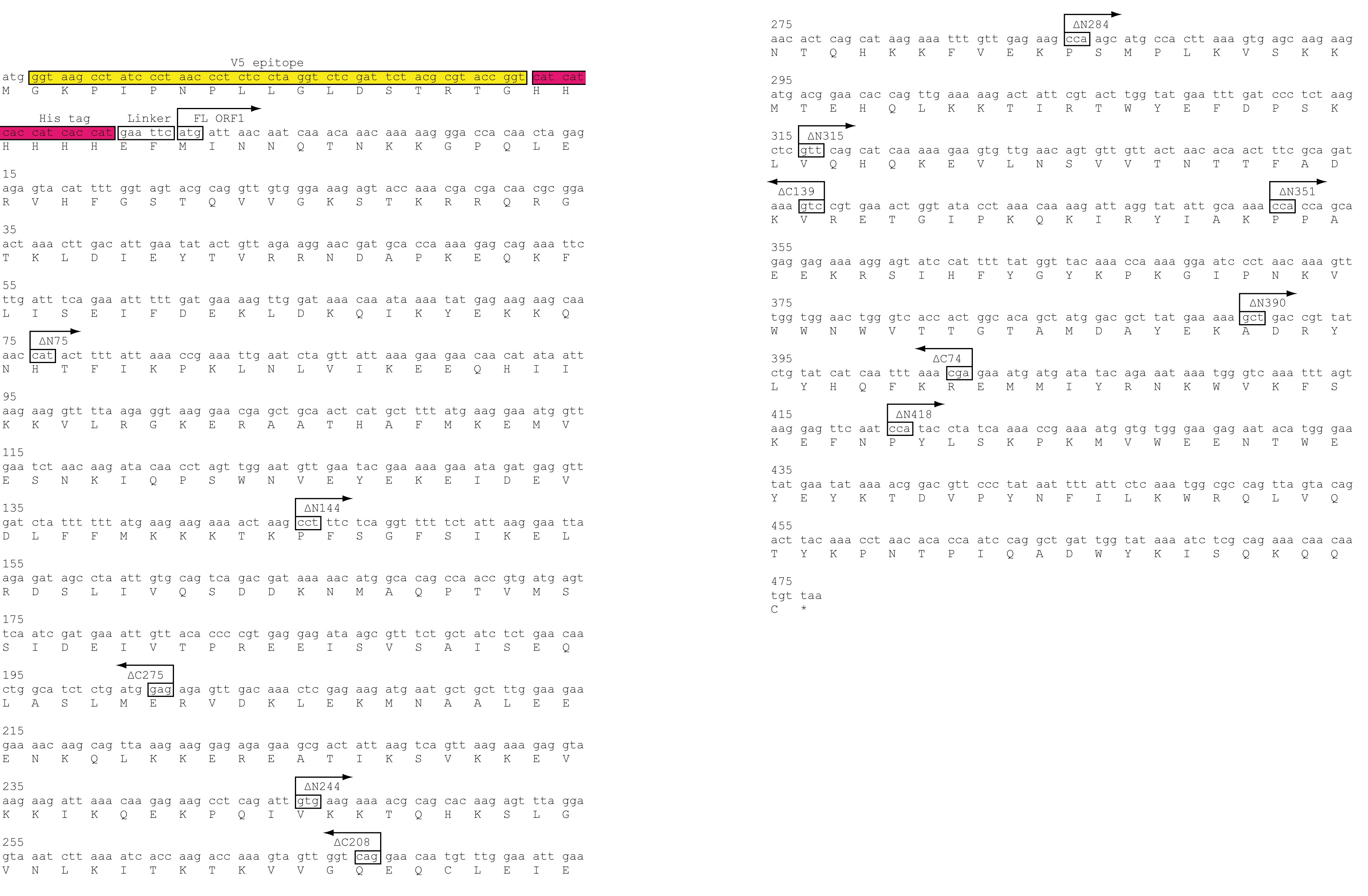

Supplement: Figure S3 — Nucleotide and protein sequence of full-length and VP1 mutants fused to V5-His tag at the N-terminus. Nucleotide and amino acid sequence of V5 epitope and Histidine (His) tagged full-length VP1 sequence (VP1FL). A linker sequence between the His tag and VP1 was created to facilitate cloning. Start and stop sites of the respective N- and C-terminal deletion mutants of VP1 are indicated. The VP1 deletion mutants were fused to the V5-His tag in an identical way as the VP1FL construct. (TIF) [file ppat.1002872.s003.tif]

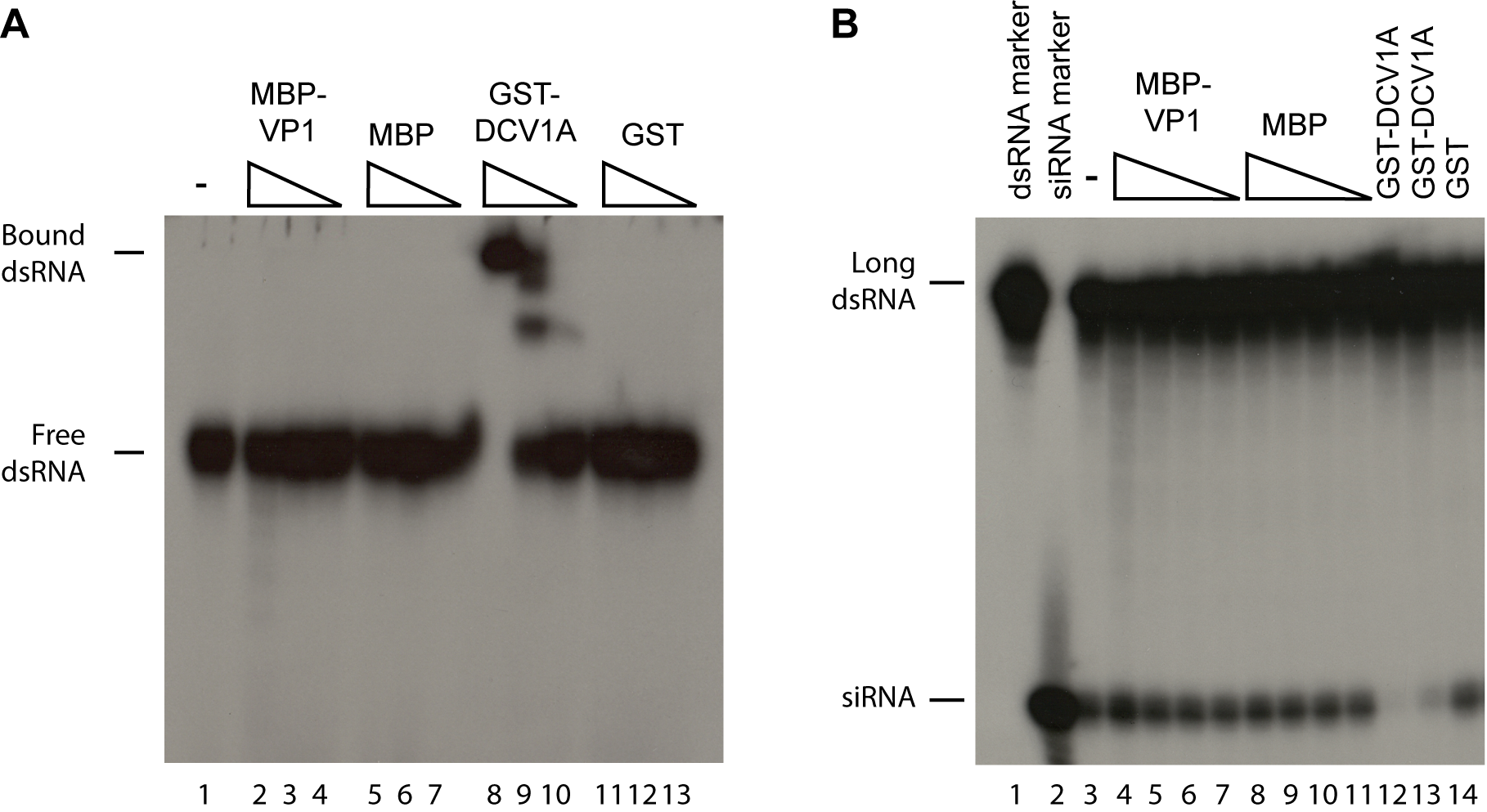

Supplement: Figure S4 — Nora virus VP1 is unable to bind long dsRNA or to interfere with Dcr-2 activity. (A) Mobility shift assay of suppressor proteins with long dsRNA. Uniformly radiolabeled long dsRNA was incubated for 30 minutes with buffer (lane 1) or recombinant MBP-VP1ΔN284 (lanes 2–4), MBP (lanes 5–7), GST-DCV 1A (lanes 8–10) or GST (lanes 11–13). Ten-fold dilutions of recombinant protein were used starting from the following concentrations: MBP-VP1ΔN284 (2 µM, lane 2), MBP (2.6 µM, lane 5), GST-DCV 1A (1 µM, lane 8), and GST (2.24 µM, lane 11). RNA mobility shifts were analyzed on an 8% native polyacrylamide gel. (B) Dicer activity in S2 cell extract in the presence of viral suppressor proteins. Uniformly radiolabeled long dsRNA was incubated in S2 cell extract for 3 hours with buffer (lane 3) or the indicated recombinant proteins. Two-fold dilutions were used for MBP-VP1ΔN284 (lanes 4–7, highest concentration 1.1 µM) and MBP (lanes 8–11, highest concentration 4.2 µM). Two independent preparations of GST-DCV 1A were used (lane 12, concentration of 0.54 µM and lane 13, concentration of 0.03 µM). GST was used at a concentration of 1.2 µM (lane 14). As size markers, dsRNA input (lane 1) and end-labelled siRNAs (lane 2) were used. Dicer products were analyzed on a 12% denaturing polyacrylamide gel. (TIF) [file ppat.1002872.s004.tif]

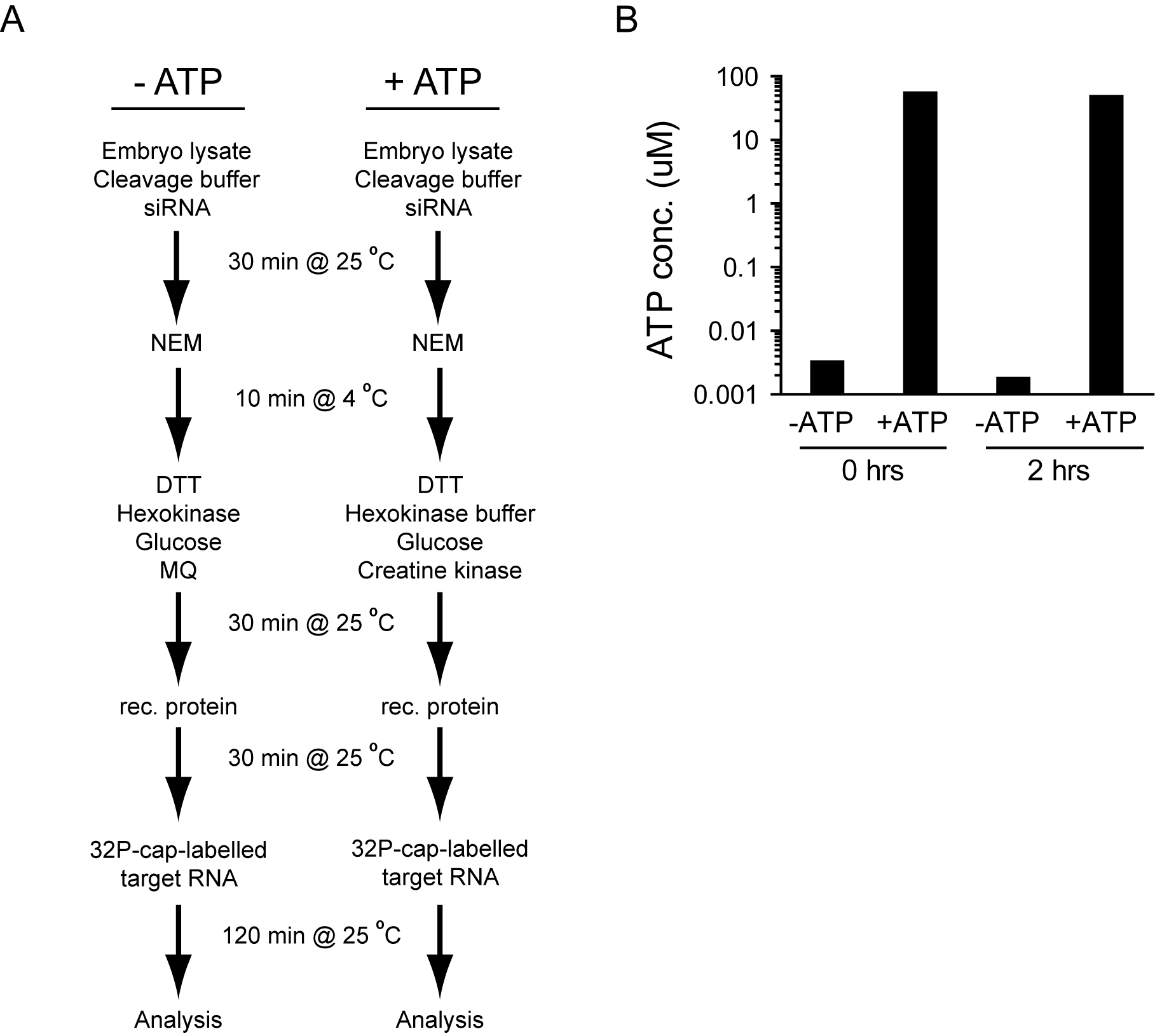

Supplement: Figure S5 — ATP depletion during Slicer assay. (A) Schematic representation of the protocol used to deplete (−ATP) or to regenerate ATP after initial depletion (+ATP) for Slicer assays of Figure 7F. For RISC loading, Drosophila embryo lysate was incubated with an siRNA for 30 minutes under standard conditions. Subsequently, N-ethylmaleimide (NEM) was added in both conditions to inhibit the ATP regenerating activity of creatine kinase. After incubating the reactions for 10 minutes on ice, DTT was added to quench the NEM in both conditions. Hexokinase, glucose, and milliQ water (MQ) were added in the –ATP protocol to deplete the pool of ATP. For the +ATP condition, Hexokinase was substituted by hexokinase buffer, and MQ was substituted for Creatine kinase to restore the ATP regenerating activity. Subsequently, the reactions were incubated for 30 minutes after which recombinant protein (rec. protein) was added. Following another 30 minutes incubation period, the 32P-cap-labelled RNA was added to the reaction, after which the incubation was continued for another 2 hours. Subsequently, reactions were analyzed on a polyacrylamide gel. (B) ATP concentrations before and after the Slicer assay under –ATP and +ATP conditions. ATP levels were measured at the moment of target RNA addition (0 hrs) or after 2 hours of incubation with target RNA. For ATP concentration measurements, recombinant protein was substituted for protein storage buffer, and target RNA was substituted for MQ. ATP levels were measured using the Celltiter-Glo Luminescent Cell Viability Assay (Promega) according to the manufacturer's protocol. (TIF) [file ppat.1002872.s005.tif]
