# Supplementary material for: Convergent Evolution of Argonaute-2 Slicer Antagonism in Two Distinct Insect RNA Viruses
Source: PLoS Pathog. 2012 Aug 16;8(8):e1002872. doi: 10.1371/journal.ppat.1002872 (PMC3420963; doi:10.1371/journal.ppat.1002872)
Supplement: Protocol S1 — Extended and supplemental methods for molecular cloning, miRNA sensor assay, clearance of Wolbachia and endogenous viruses from fly stocks, production of recombinant proteins in E. coli , production of radio-labeled probes and target RNA, Dicer and Slicer assays. (DOC) [file ppat.1002872.s006.doc]

**Protocol S1**

**Extended Experimental Procedures and Supplemental Methods**

**Molecular cloning**

To construct plasmids encoding C-terminal V5 epitope tagged proteins of Nora virus, cDNA prepared from Nora virus infected flies was amplified using primers 5’-AGT GGT ACC AAC ATG ATT AAC AAT CAA ACA AAC and 5’-GGT GGG CCC TTG ACA TTG TTG TTT CTG CG for ORF1, primers 5’-AGT GGT ACC AAC ATG TTA ATT GAA GCT TTC ATC and 5’-GGT GGG CCC TCC AAG ATC TCC TCT TTT AAT G for ORF2, primers 5’-AGT GGT ACC AAC ATG GCA TTA AAA GAG GAG ATC and 5’-GGT GGG CCC TTG CAT AGA GTC ATA AAT TAC for ORF3, and primers 5’-AGT GGT ACC AAC ATG CAG AAT CCA ACA CAA ACC and 5’-GGT GGG CCC CTG CTG CCT CAC GGA AGG GAA for ORF4. Amplified products were cloned as *Kpn*I and *Apa*I fragments into pAc5.1-V5-His-A (Invitrogen).

For the expression of VP1 mutants tagged at the N-terminus with the V5-His epitopes, the pAc5.1-V5-His-Ntag plasmid was constructed. This plasmid was created by annealing and cloning the oligonucleotides 5’-CAA CAT GGG TAA GCC TAT CCC TAA CCC TCT CCT AGG TCT CGA TTC TAC GCG TAC CGG TCA TCA TCA CCA TCA CCA TG and 5’-AAT TCA TGG TGA TGG TGA TGA TGA CCG GTA CGC GTA GAA TCG AGA CCT AGG AGA GGG TTA GGG ATA GGC TTA CCC ATG TTG GTA C into the *EcoR*I and *Kpn*I restriction sites of pAc5.1-V5-His-A. The sequences of all VP1 deletion mutants were cloned into pAc5.1-V5-His-Ntag using the *EcoR*I and *Sac*I restriction sites. For mutant sequences see supplemental Figure S3.

**miRNA sensor assay**

The miRNA sensor assay and its plasmids were described previously [1,2]. Briefly, 5x10^4^ S2 cells were seeded per well in a 96-well plate one day before transfection. Subsequently, the cells were transfected with 54.5 ng suppressor plasmid, 6.8 ng pMT-Fluc-par6, 1.6 ng pMT-Ren, and 2.7 ng pMT-miR1 or pMT-miR12 using the Effectene transfection reagent (Qiagen) according to the manufacturer’s protocol. To knockdown *AGO1* or *AGO2* expression, 5.4 ng of dsRNA was cotransfected with the plasmids. Expression of the reporter constructs was induced with CuSO_4_ at 48 hrs post transfection and luciferase activities were measured at 72 hrs post transfection.

**Clearance of *Wolbachia* and endogenous viruses from fly stocks**

Fly stocks used for Sindbis virus infection and preparation of embryo lysates were cleared from endogenous viruses by collecting eggs on apple-juice agar plates, followed by a treatment with 50% household bleach for 5 minutes. Subsequently, the bleached eggs were washed three times in a large volume of water, after which they were transferred to clean vials containing standard fly food. After culturing the fly stocks for two generations we confirmed the absence of Nora virus and Drosophila C virus (DCV) by RT-PCR. Fly stocks were then cleared from *Wolbachia* infection by raising the flies for two generations on standard fly food supplemented with 0.05 mg/mL tetracycline hydrochloride (Sigma). To verify the clearance of *Wolbachia* infection, PCR amplification was performed with *Wolbachia* specific primers on DNA extracts of adult flies, as described earlier [3].

**Production of recombinant proteins in *E. coli***

To fuse the VP1^ΔN284^ protein to the C-terminus of maltose binding protein (MBP), the coding sequence of the VP1^NΔ284^ mutant was cloned as an *EcoR*I-*Sal*I fragment into the pMal-C2X vector (New England Biolabs). The resulting pMal-C2X-VP1^NΔ284^ and parental pMal-C2X plasmids were transformed into the *E.coli* BL21 (DE3) strain. Expression of the recombinant fusion proteins was induced at 1.0 OD_600_ by adding 0.2 mM IPTG followed by incubation at 37^○^C for 3 hours. MBP and MBP-VP1^NΔ284^ fusion proteins were purified using amylose resin (New England Biolabs) according to the manufacturer’s protocol.

The coding sequence of CrPV 1A (amino acids 1-148) was amplified using primers 5’- CGG GAA TTC ATG TCT TTT CAA CAA ACA AAC AAC and 5’- AGA GTC GAC TTA GAA GGC TCT GCA TT and cloned into the pGEX-4T-1 plasmid (GE healthcare) as an *EcoR*I-*Sal*I fragment. After transformation of the *E. coli* BL21 (DE3) strain with the resulting pGEX-CrPV 1A plasmid, expression was induced at 1.0 OD_600_ using 0.2 mM IPTG. Protein production was allowed to continue overnight at 20^○^C. The GST-CrPV 1A fusion protein was purified using glutathione sepharose 4 fast flow (GE healthcare) according to the manufacturer’s protocol. GST (pGEX-4T-1) and GST-DCV 1A (pGEX-DCV 1A) fusion proteins were purified using the same method, after induction of protein expression at 37^○^C for 3 hours [4].

**Radioactively labeled probes and target RNA**

Uniformly radio-labeled 113 bp long dsRNA was generated by *in vitro* transcription in the presence of α-[^32^P]-UTP using a T7 promoter flanked firefly luciferase (GL3) PCR product as a template. T7 promoter flanked PCR products were generated with primers 5’-TAA TAC GAC TCA CTA TAG GGA GAT ATG AAG AGA TAC GCC CTG GTT and 5’-TAA TAC GAC TCA CTA TAG GGA GAA TAG CTT CTG CCA ACC GAA C. Unincorporated nucleotides were removed using a G-25 sephadex column (Roche) followed by purification of the dsRNA from an 8% polyacrylamide gel. Gl3 siRNAs (Dharmacon) were ^32^P end-labeled using T4 polynucleotide kinase (Roche) after which unincorporated nucleotides were removed using a G-25 sephadex column (Roche).

To generate target RNA for the Slicer assay, a 492 bp region of the GL3 luciferase gene was PCR amplified using the primers 5’-TAA TAC GAC TCA CTA TAG GGA GAA TGG AAG ACG CCA AAA ACA T and 5’-CAT CGA CTG AAA TCC CTG GT. The GL3 PCR product was used as a template for *in vitro* transcription using the Ampliscribe T7 flash transcription kit (Epicentre). After purification from an 8% urea-polyacrylamide gel, the RNA was cap-radiolabeled with the Scriptcap m^7^G capping system (Epicentre) according to the manufacturer’s protocol. The capped RNA was purified from an 8% Urea-polyacrylamide gel before use in the Slicer assay.

**Dicer assay**

Dicer assays were performed in a final volume of 12 µL containing 4µL S2 cell extract, 3 µL dicer buffer, 1 µL uniformly labeled dsRNA (200 cps), and 4 µL purified recombinant protein. Dicer buffer contained 0.175 µg/µL creatine kinase (Roche), 16.7 mM DTT, 0.02 mg/µL creatine monophosphate (Roche), 3.3 mM MgAc, 50 mM Hepes-KOH, 33.3% glycerol, 0.67 U/µL RNasin (Roche), and 3.3 mM ATP. Reactions were incubated for 3 hrs at 27^○^C after which they were deproteinized by proteinase K and phenol extracted [5]. After precipitation, the RNA was dissolved in Ambion loading buffer II and loaded on a 12% denaturing polyacrylamide gel. Dicer products were visualized by exposing the polyacrylamide gel to a Kodak Biomax XAR film.

**Slicer assay**

*Drosophila* embryo lysates were produced from *w^1118^* flies as described [5]. Slicer reactions contained 5 µL embryo lysate, 3 µL cleavage buffer, 100 nM siRNA, 0.3 µM recombinant protein, and 1 µL capped target RNA (~1000 cps) in a final volume of 11 µL. The GL3 siRNA (Dharmacon) was used to induce cleavage of the firefly luciferase target RNA, whereas the negative control siRNA (Qiagen) was used as a negative control. After assembly of the reaction, samples were incubated for 2 hours at 25^○^C. Samples were then treated with proteinase K, extracted with phenol, and precipitated as described [5]. Precipitated RNA was dissolved in Loading buffer II (Ambion) and analyzed on an 8% urea-polyacrylamide gel. Slicer products in ATP depleting conditions were analyzed on a 6% urea-polyacrylamide gel. Kodak Biomax XAR films were used to visualize the radioactive Slicer products.

**References**

1. Eulalio A, Rehwinkel J, Stricker M, Huntzinger E, Yang SF et al. (2007) Target-specific requirements for enhancers of decapping in miRNA-mediated gene silencing. Genes Dev 21: 2558-2570.

2. Schnettler E, Hemmes H, Huismann R, Goldbach R, Prins M et al. (2010) Diverging affinity of tospovirus RNA silencing suppressor proteins, NSs, for various RNA duplex molecules. J Virol 84: 11542-11554.

3. Teixeira L, Ferreira A, Ashburner M (2008) The bacterial symbiont Wolbachia induces resistance to RNA viral infections in Drosophila melanogaster. PLoS Biol 6: e2.

4. Van Rij RP, Saleh MC, Berry B, Foo C, Houk A et al. (2006) The RNA silencing endonuclease Argonaute 2 mediates specific antiviral immunity in Drosophila melanogaster. Genes Dev 20: 2985-95.

5. Haley B, Tang G, Zamore PD (2003) In vitro analysis of RNA interference in Drosophila melanogaster. Methods 30: 330-6.
