# Supplementary material for: Convergent Evolution of Argonaute-2 Slicer Antagonism in Two Distinct Insect RNA Viruses
Source: PLoS Pathog. 2012 Aug 16;8(8):e1002872. doi: 10.1371/journal.ppat.1002872 (PMC3420963; doi:10.1371/journal.ppat.1002872)
Supplement: Text S1 — Nora virus VP1 is unable to suppress the miRNA pathway. (DOC) [file ppat.1002872.s007.doc]

**Text S1**

**Nora virus VP1 is unable to suppress the miRNA pathway**

Several plant virus RNAi suppressors influence the miRNA pathway, thereby inducing strong developmental defects in transgenic plants that express RNAi suppressors during development [1,2]. This effect may be due to convergence of the antiviral RNAi and miRNA pathways on Argonaute-1 (AGO1) in plants. In *Drosophila*, the miRNA and siRNA pathways are parallel pathways. Nevertheless, there is crosstalk between these pathways with miRNA and miRNA-star sequences being loaded into AGO2 and, conversely, with siRNAs being loaded into AGO1 [3,4]. To determine whether VP1 suppresses the miRNA pathway, we used a miRNA sensor assay in S2 cells (Protocol S1). In this assay, an Fluc reporter containing the 3’UTR of the *Drosophila* *par6* gene (Fluc-par6), a target for miRNA1, is co-transfected with a plasmid expressing the primary miRNA1 (pri-miR1), or a control plasmid expressing pri-miR12 [5,6]. Co-transfection of pri-miR1 led to specific silencing of the Fluc-par6 gene (Figure S1). We verified whether the reporter was suppressed in an *AGO1* dependent manner, by cotransfection of dsRNA targeting *AGO1* or, as a control, *AGO2*. As expected, the miRNA reporter assay monitors the canonical miRNA pathway, since knock-down of the *AGO1* gene by dsRNA led to de-repression of Fluc-par6 expression (although this did not reach statistical significance, p=0.09). In contrast, co-transfection of AGO2 dsRNA did not lead to de-repression, but even enhanced silencing of the miRNA reporter, perhaps reflecting more efficient AGO1 loading under conditions in which AGO2 is depleted. Expression of Nora virus VP1 did not de-repress the Fluc-par6 construct, indicating that VP1 does not suppress the miRNA pathway. Similarly, VP1 did not affect silencing of a miRNA sensor consisting of a luciferase construct containing two perfect complementary target sites for the endogenous miR2 in its 3’UTR (data not shown) [7]. In addition, transgenic flies expressing VP1 driven by a strong ubiquitous promoter (Tubulin-Gal4) are viable and fertile, lending further support to the conclusion that VP1 does not inhibit miRNA biogenesis and function (data not shown).

**References**

1. Chapman EJ, Prokhnevsky AI, Gopinath K, Dolja VV, Carrington JC (2004) Viral RNA silencing suppressors inhibit the microRNA pathway at an intermediate step. Genes Dev 18: 1179-1186.

2. Jay F, Wang Y, Yu A, Taconnat L, Pelletier S et al. (2011) Misregulation of AUXIN RESPONSE FACTOR 8 underlies the developmental abnormalities caused by three distinct viral silencing suppressors in Arabidopsis. PLoS Pathog 7: e1002035.

3. Ghildiyal M, Xu J, Seitz H, Weng Z, Zamore PD (2010) Sorting of Drosophila small silencing RNAs partitions microRNA* strands into the RNA interference pathway. RNA 16: 43-56.

4. Okamura K, Liu N, Lai EC (2009) Distinct mechanisms for microRNA strand selection by Drosophila Argonautes. Mol Cell 36: 431-444.

5. Eulalio A, Rehwinkel J, Stricker M, Huntzinger E, Yang SF et al. (2007) Target-specific requirements for enhancers of decapping in miRNA-mediated gene silencing. Genes Dev 21: 2558-2570.

6. Schnettler E, Hemmes H, Huismann R, Goldbach R, Prins M et al. (2010) Diverging affinity of tospovirus RNA silencing suppressor proteins, NSs, for various RNA duplex molecules. J Virol 84: 11542-11554.

7. Van Rij RP, Saleh MC, Berry B, Foo C, Houk A et al. (2006) The RNA silencing endonuclease Argonaute 2 mediates specific antiviral immunity in Drosophila melanogaster. Genes Dev 20: 2985-95.
